# Qianyang Yuyin Granules for vascular damage in mild-to-moderate hypertensive patients: a systematic review with meta-analysis and trial sequential analysis

**DOI:** 10.3389/fphar.2025.1612508

**Published:** 2025-11-19

**Authors:** Shidian Zhu, Qiannong Wu, Yanlin Liu, Fuming Liu

**Affiliations:** 1 Affiliated Hospital of Nanjing University of Chinese Medicine, Jiangsu Province Hospital of Chinese Medicine, Nanjing, China; 2 The First Clinical Medical College, Nanjing University of Chinese Medicine, Nanjing, China; 3 Affiliated Hospital of Integrated Traditional Chinese and Western Medicine, Nanjing University of Chinese Medicine, Nanjing, China

**Keywords:** hypertension, vascular damage, Qianyang Yuyin granules, meta-analysis, trial sequential analysis

## Abstract

**Objective:**

The purpose is to evaluate the clinical efficacy and safety of Qianyang Yuyin Granules in the treatment of vascular damage in essential hypertension systematically.

**Methods:**

Relevant literature databases include CNKI, Wanfang Data, VIP, CBM, PubMed, Web of Science, Cochrane Library. DeepSeek-V3 was used for AI-assisted retrieval. The time limit for searching was from the establishment of the database to 15 April 2025. Eligible randomized controlled clinical trials were screened according to inclusion and exclusion criteria. Publication bias assessment and quality evaluations were analysed by using the Cochrane Handbook and meta-analysis was performed by using RevMan 5.3 software. Finally, using Trial Sequential Analysis 0.9Beta software to perform the trial sequential analysis.

**Results:**

21 studies were finally included, with a total sample of 1,419 cases, 728 cases in the experimental group and 691 cases in the control group. Meta-analysis suggested that the trial group using Qianyang Yuyin Granules was effective in lowering PWV-BS [MD = −0.58, 95%CI: (−0.88, −0.29), *P* < 0.0001], PWV-ES [MD = −0.79, 95%CI: (−1.22, −0.36), *P* = 0.0003] in the elderly population with essential hypertension (≥60 years old), lowering inflammatory factor-related indexes including CRP [SMD = −0.37, 95%CI: (−0.58, −0.16), *P* = 0.0005] and TNF-α [SMD = −0.44, 95%CI: (−0.70, −0.18), *P* = 0.001], lowering systolic blood pressure [MD = −3.86, 95%CI: (−5.33, −2.39), *P* < 0.00001] and diastolic blood pressure [MD = −2.24, 95%CI: (−3.39, −1.10), *P* = 0.0001] were all superior to the control group that only used basic treatment and the differences were statistically significant; The incidence rate of adverse reactions in the experimental group was not statistically significant when compared with that of the control group [OR = 0.71, 95%CI: (0.26, 1.93), *P* = 0.51]. The sequential analysis further suggested that the addition of Qianyang Yuyin Granules to reduce PWV and CRP in peripheral blood had a precise role.

**Conclusion:**

The addition of Qianyang Yuyin Granules in the treatment of vascular damage in mild-to-moderate essential hypertension can improve the clinical efficacy without increasing the adverse effects of conventional drugs, and has further clinical application value. However, further research with larger scale and stricter quality control is needed.

**Systematic review registration:**

https://www.crd.york.ac.uk/PROSPERO/, identifier CRD42025641482.

## 1 Introduction

Essential hypertension, a vascular disease primarily characterized by the elevation of systemic arterial blood pressure, frequently causes damage to vital target organs including the heart, brain, and kidneys. Regarding vascular damage, patients exhibit evident vascular endothelial function impairment and reduced carotid artery elastic function in the early stage. This situation further facilitates the development of atherosclerosis in patients (Xiong et al., 2019) and hastens the process of damage to other target organs. Numerous studies have demonstrated that endothelial cell activation and dysfunction in hypertension are closely associated with inflammation. The levels of inflammatory factors, such as C-reactive protein (CRP) and tumor necrosis factor-α (TNF-α), are positively correlated with hypertension (Fu et al., 2022). For the diagnosis of vascular damage, measuring the ultra fast imaging pulse wave velocity (UFPWV) can reflect arterial stiffness. UFPWV is sensitive, convenient, and rapid in the early stage. This technique evaluates the degree of arterial elasticity by collecting the pulse wave velocity (PWV) at two phases of a cardiac cycle, namely, the beginning of systole (BS) and the end of systole (ES).

Regarding treatment, current conventional Western antihypertensive drugs, such as angiotensin-converting enzyme inhibitors (ACEI), angiotensin II receptor blockers (ARB), and calcium channel blockers (CCB), have been demonstrated by research to have a certain effect in ameliorating vascular damage (Ghiadoni L, 2016). These medications not only lower blood pressure effectively but also alleviate the structural and functional damage to blood vessels caused by hypertension. By reducing such vascular impairment, they ultimately lower the risk of cardiovascular and cerebrovascular complications, making them the preferred choice for hypertensive patients with vascular disease. However, their efficacy still remains limited. The integration of traditional Chinese and Western medicine in treatment represents the current mainstream approach in China and has demonstrated favorable clinical efficacy. Numerous studies have indicated that traditional Chinese medical therapies, including classic prescriptions, Chinese patent medicines and acupuncture, can steadily reduce blood pressure, ameliorate related risk factors, and safeguard target organs (Xiong et al., 2023).

Qianyang Yuyin Granules (QYYYG), a renowned hospital preparation of Jiangsu Province Hospital of Chinese Medicine, originates from Jiangya Yishen Prescription formulated by Professor Tang Shuhua, a distinguished veteran traditional Chinese medicine practitioner. Subsequently, it was improved by Professor Fang Zhuyuan and effectively applied. This preparation is composed of six traditional Chinese medicinal herbs, specifically Herba Bidentis Bipinnatae (*Bidens pilosa L.* , Gui Zhen Cao), Scrophulariae Radix (*Scrophularia ningpoensis Hemsl.* , Xuan Shen), Alismatis Rhizoma [*Alisma plantago-aquatica subsp. Orientale (Sam.) Sam.* , Ze Xie], Polygoni Multiflori Radix [*Reynoutria multiflora (Thunb.) Moldenke*, Zhi He Shou Wu], Corni Fructus (*Cornus officinalis Siebold & Zucc.* , Shan Zhu Yu), Cyathulae Radix (*Cyathula officinalis K.C.Kuan*, Chuan Niu Xi). Validated by clinical practice and multiple randomized controlled trials, QYYYG has demonstrated efficacy in effectively reducing blood pressure and alleviating vascular damage, particularly in patients with grade 1 and grade 2 hypertension. Compared with conventional Western medicine treatments, QYYYG shows superior therapeutic effects and has been widely applied in clinical practice. However, at present, there is still a lack of validation from large-sample, multi-center, and high-quality clinical trials. Furthermore, there remain controversies in the results of several small-sample clinical trials concerning whether QYYYG can effectively sustain blood pressure reduction, mitigate vascular damage, and regarding potential safety concerns.

**FIGURE 1:**
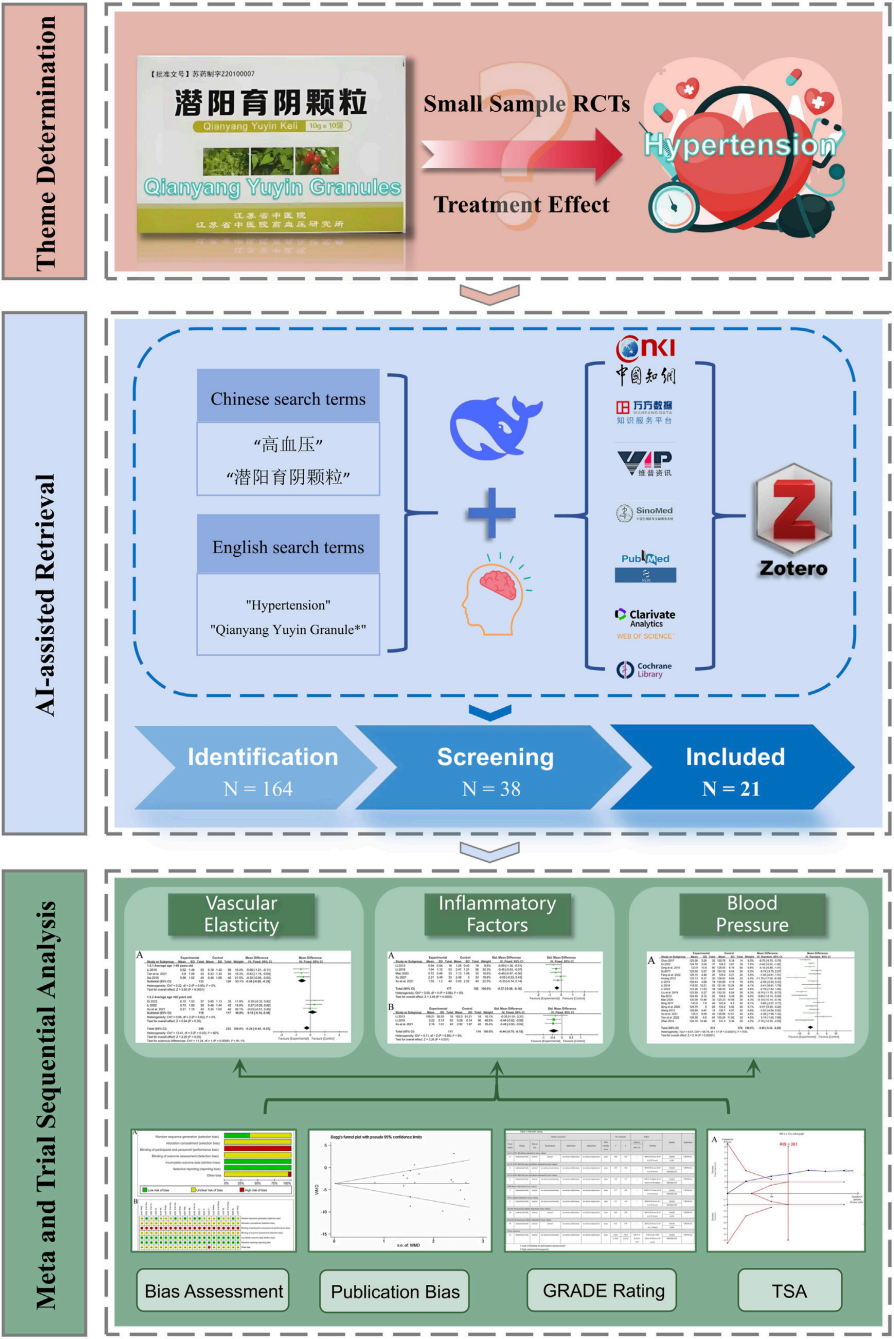
The overall flow chart.

This study aims to conduct a systematic evaluation of the clinical efficacy and safety of QYYYG in treating vascular damage associated with essential hypertension via Meta-analysis and sequential analysis. The objective is to offer a more optimal integrated traditional Chinese and Western medicine treatment strategy for patients with hypertension. The overall flow chart of this study is shown in Figure 1.

## 2 Materials and methods

### 2.1 Study registration

This study was conducted and reported in accordance with the Preferred Reporting Project (PRISMA) guidelines for systematic reviews and meta-analyses (Page et al., 2021). The study protocol was registered (CRD42025641482) in the International prospective register of systematic reviews (PROSPERO).

### 2.2 Retrieval strategy

To comprehensively retrieve relevant information, databases such as CNKI, Wanfang Data, VIP, CBM, PubMed, Web of Science, and Cochrane Library were systematically searched. The objective was to identify published randomized controlled trials (RCTs) on the application of QYYYG in treating hypertensive vascular damage. The retrieval time range extended from the inception of each database to 15 April 2025. We combine the large language model to carry out AI-assisted retrieval. Adopt the retrieval keywords obtained by individual retrieval, and ask the DeepSeek-V3 (https://www.deepseek.com/) according to different databases. It is required to synthesize the recall rate and precision rate, generate the specific logical relationship and retrieval formula, and use the recommended retrieval formula for database retrieval. Finally, the retrieved documents are imported into Zotero document management software for management and analysis. For more details on the retrieval strategy, please refer to the Supplementary Material.

### 2.3 Inclusion criteria

#### 2.3.1 Participants (P)

The study subjects were adult patients with grade 1 or 2 essential hypertension, who met the traditional Chinese medicine syndrome differentiation criteria of liver fire hyperactivity or yin deficiency leading to yang hyperactivity. The diagnostic criteria were based on the “Guidelines for the Prevention and Treatment of Hypertension in China (2018 Revision)” (Writing Group of 2018 Chinese Guidelines for the Management of Hypertension, 2019).

#### 2.3.2 Intervention (I) and comparison (C)

For the control group, the basic treatment protocol was implemented. Based on lifestyle modifications, lipid-regulating measures, etc., the primary recommended treatment plan in the “Guidelines for the Prevention and Treatment of Hypertension in China (2018 Revision)” (Writing Group of 2018 Chinese Guidelines for the Management of Hypertension, 2019) was followed. Calcium channel blockers (CCB), angiotensin-converting enzyme inhibitors/angiotensin II receptor blockers (ACEI/ARB), or their combinations were utilized. For the experimental group, Qianyang Yuyin Granules (with a specification of 10 g per bag, Patent Authorization Number: ZL201010205024.2) were added to the treatment measures already applied to the control group.

#### 2.3.3 Outcome (O)

1. Indicators related to vascular elasticity (PWV-BS, PWV-ES): Aixplorer ultrasound diagnostic system (Supersonic Imagine, France) was used, with the built-in UFPWV examination technology (probe model: SL10-2, frequency range: 1.0–2.0 MHz, examination mode: Vascular/Carotid) to measure the subjects’ PWV-BS and PWV-ES. All subjects were placed in a supine position without a pillow, with their necks fully exposed, and measurements were taken separately on the left and right sides. Starting from the aortic arch origin and the innominate artery bifurcation, scans were performed first in transverse section and then in longitudinal section under the gray-scale imaging mode to observe the common carotid artery (CCA) and internal/external carotid arteries. When scanning the intima of the anterior and posterior vessel walls, longitudinal exploration of the long-axis section of the bilateral CCA was conducted to ensure clear imaging. The probe was placed perpendicular to the maximum long-axis section of the CCA, and a section that clearly displayed the adventitia of the CCA anterior wall was selected. The carotid bulb and plaques were avoided, and the probe was kept stationary for 2 s to measure PWV. The variation range (Δ±) was controlled within 10% of the BS or ES value to ensure stable data collection.
2. Indicators related to inflammatory factors (CRP, TNF-α): before and after treatment, fasting venous blood samples were collected from patients in the early morning and sent to the clinical laboratory for testing.
3. Blood pressure levels (systolic blood pressure, diastolic blood pressure); under resting conditions, the patient’s brachial artery office blood pressure was measured three times, with a 2-min interval between each measurement, and the average value was recorded.
4. Safety indicators: including blood routine, urine routine, electrocardiogram, liver and kidney function, etc.

#### 2.3.4 Study design (S)

All included studies were randomized controlled trials (RCTs).

### 2.4 Exclusion criteria

Studies with duplicate publication or duplicate research data; 2) Studies with incomplete research data and lacking indicators related to vascular damage; 3) Non-randomized controlled trial studies such as reviews, clinical experiences, and animal experiments; 4) Studies whose subjects include patients with grade 3 hypertension, secondary hypertension, or those with severe target organ functional damage, or those complicated with severe diseases of other systems.

### 2.5 Quality evaluation criteria

The Cochrane Risk of Bias Assessment Tool was employed to assess the quality of the studies across seven dimensions: random sequence generation, allocation concealment, blinding of participants and research staff, blinding of outcome assessment, completeness of outcome data, selective reporting, and other potential sources of bias. Two researchers independently and blindly carried out the evaluation and cross-checked the results with each other. In the event of a disagreement, the matter will be referred to a third researcher for discussion and resolution. Subsequently, the final bias proportion diagram and quality assessment diagram will be generated. In addition, GRADEpro is used to evaluate the evidence quality of different indicators.

### 2.6 Data extraction

In accordance with the pre-determined retrieval strategy, two researchers independently and in a blinded manner utilized Zotero software to construct a relevant database for the purposes of studies screening and data extraction. Firstly, through browsing the titles and abstracts, exclude the studies that are evidently irrelevant to QYYYG or are duplicates. Secondly, read the full text meticulously and decide on the included studies based on the inclusion and exclusion criteria. Thirdly, after completing the above steps, cross check the inclusion results and reasons provided by the two researchers, and use Kappa statistic to evaluate consistency. In the event of any disagreement, the issue will be referred to a third researcher for discussion and resolution. Finally, Excel spreadsheets were used for data extraction. The data extraction items included: study title, first author’s name, publication year, sample size, mean age, gender distribution, intervention strategies, treatment duration, outcome measures, among others.

### 2.7 Statistical analysis

RevMan 5.3 and TSA 0.9Beta software were used to perform Meta-analysis and trial sequential analysis on the collected research data. The Q-test and I^2^-test were employed to assess the heterogeneity. If the P-value is greater than 0.1 and the I^2^ value is less than 50%, it suggests that the heterogeneity is low, and the fixed-effect model should be applied for the analysis. Conversely, if the heterogeneity is high, the random-effect model will be utilized for the analysis. Subgroup analysis will be conducted based on the overall characteristics of the study to identify the sources of heterogeneity. Sensitivity analysis will be conducted using STATA 12.0 software. Meanwhile, Begg’s test and Egger’s test will be applied to check for the presence of publication bias in this study. Finally, the TSA 0.9Beta software will be used to conduct a further analysis of the studies with low heterogeneity, thereby enhancing the credibility of the results.

## 3 Results

### 3.1 Results of studies filtering

**FIGURE 2:**
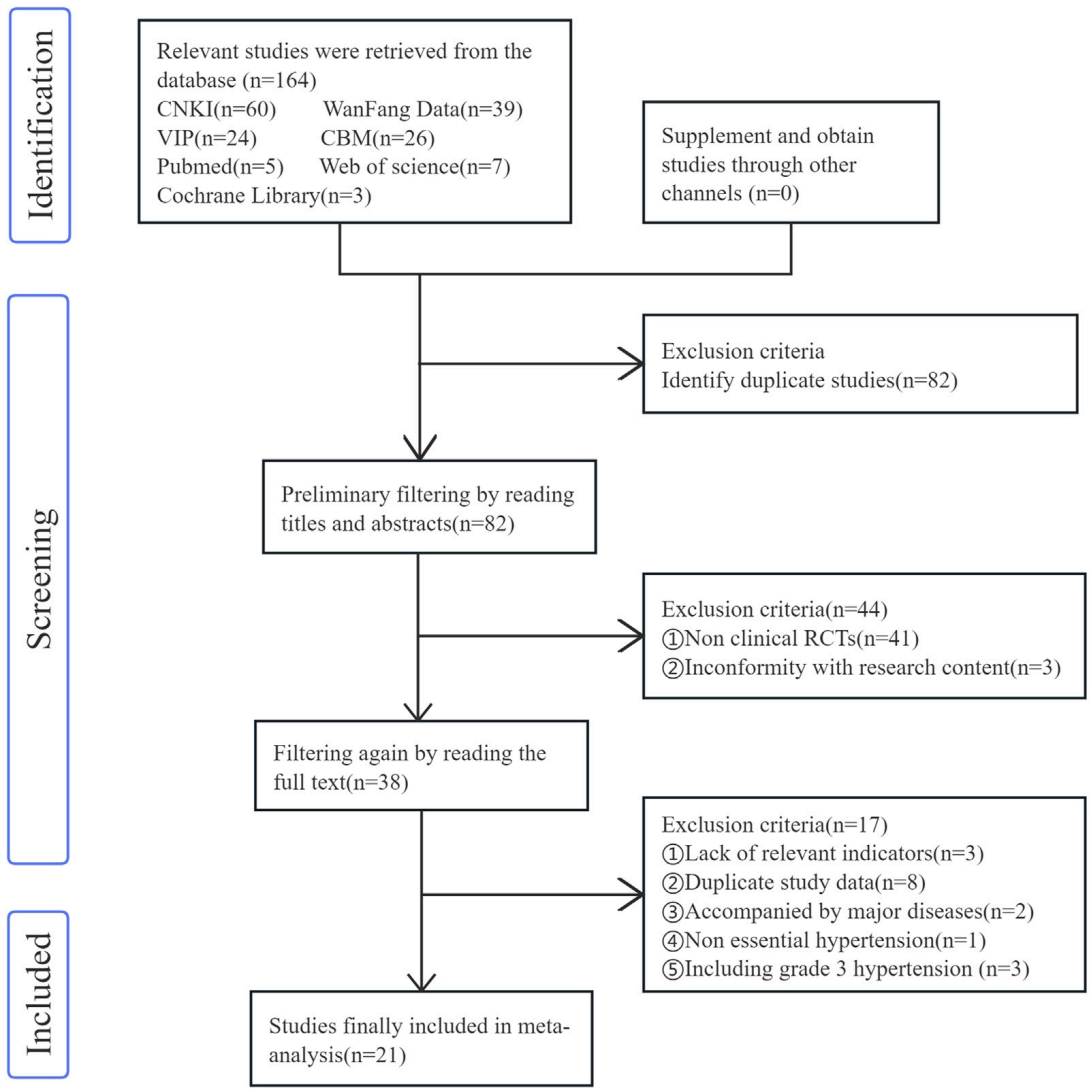
Studies filtering process.

In accordance with the pre-formulated retrieval strategy, a total of 164 relevant studies were initially retrieved. Among them, 82 articles were identified as duplicates and thus excluded. Two researchers achieved excellent consistency in screening literature titles and abstracts (Kappa = 0.927) and good consistency in full-text screening (Kappa = 0.837). Discrepancies in literature selection were resolved through group discussions involving a third researcher, and 21 studies (Li, 2022; Dinala Jialiken, 2022; Xu et al., 2021; Tan et al., 2021; Li, 2018; Xia, 2018; Li, 2013; Xu, 2021; Mao, 2020; Yan et al., 2022; Huang, 2012; Wang, 2013; Liu et al., 2019; Chen, 2017; Du, 2017; Zhao, 2014; Ma, 2013; Fang et al., 2022a; Ning, 2017; Ding et al., 2015; Qin, 2020) were ultimately included in the analysis. The publication years of these studies ranged from 2013 to 2022. The combined sample size of all the included studies was 1,419 cases, with 728 cases in the experimental group and 691 cases in the control group. The detailed filtering process is illustrated in Figure 2.

### 3.2 Included studies table

A total of 21 eligible randomized controlled studies were included, all of which were Chinese-language articles. The basic data are shown in Table 1.

**TABLE 1:** Basic characteristics of the included studies.

Study (Year)	Sample size (T/C, n)	Age ( $\bar{X}$ ± *s*, year)		Gender (Male/Female, n)		Intervention		Duration	Outcome
		T	C	T	C	T	C		
Li (2022)	39/42	54.46 ± 8.07	55.52 ± 8.81	16/23	22/20	BT + QYYYG	Basic treatment (amlodipine)	12w	①③④
Dinala Jialiken (2022)	37/35	49.66 ± 11.18^a^	47.42 ± 9.28^a^	22/15	17/18	BT + QYYYG	Basic treatment	12w	①③④
Xu et al. (2021)	40/40	51.56 ± 10.87	53.58 ± 10.47	25/15	19/21	BT + QYYYG	Basic treatment (amlodipine)	12w	①②③④
Tan et al. (2021)	40/40	59.98 ± 7.88	62.65 ± 6.97	21/19	24/16	BT + QYYYG	Basic treatment (amlodipine)	12w	①④
Li (2018)	53/56	60.83 ± 12.02	60.09 ± 11.69	27/26	30/26	BT + QYYYG	Basic treatment (CCB, ACEI/ARB)	8w	①②③④
Xia (2018)	40/40	67.28 ± 6.50	68.10 ± 5.49	19/21	21/19	BT + QYYYG	Basic treatment (amlodipine, valsartan)	6w	①④
Li (2013)	18/18	68.22 ± 6.26	66.94 ± 7.13	6/12	6/12	BT + QYYYG	Basic treatment (nifedipine, enalapril)	8w	②③④
Xu (2021)	33/33	68.18 ± 7.04	70.09 ± 8.55	20/13	18/15	BT + QYYYG	Basic treatment (CCB, ACEI/ARB)	24w	②④
Mao (2020)	33/35	45.42 ± 9.09	50.17 ± 11.96	22/11	20/15	BT + QYYYG	Basic treatment (CCB, ACEI/ARB)	8w	②③④
Yan et al. (2022)	64/33	66.95 ± 6.55	66.69 ± 4.09	22/42	14/19	BT + QYYYG	Basic treatment	24w	③④
Huang (2012)	20/20	62.50 ± 5.50	61.40 ± 6.80	8/12	9/11	BT + QYYYG	Basic treatment (nifedipine, enalapril)	8w	③④
Wang (2013)	18/20	54.56 ± 11.54	54.25 ± 11.57	8/10	10/10	BT + QYYYG	Basic treatment (nifedipine, enalapril)	8w	③④
Liu et al. (2019)	30/30	63.20 ± 7.27	64.30 ± 6.32	16/14	18/12	BT + QYYYG	Basic treatment (amlodipine, valsartan)	24w	③
Chen (2017)	25/14	51.10 ± 10.44	49.00 ± 11.82	NA	NA	BT + QYYYG	Basic treatment (CCB, ACEI/ARB)	24w	③
Du (2017)	30/30	62.50 ± 11.86	64.80 ± 11.20	15/15	15/15	BT + QYYYG	Basic treatment (amlodipine, valsartan)	24w	③
Zhao (2014)	20/20	66.20 ± 6.29	69.05 ± 5.94	10/10	11/9	BT + QYYYG	Basic treatment (nifedipine, valsartan)	8w	③
Ma (2013)	20/20	49.00 ± 11.82	55.10 ± 10.44	11/9	10/10	BT + QYYYG	Basic treatment (nifedipine, enalapril)	6w	③
Fang et al. (2022b)	20/20	73.00 ± 4.71	73.85 ± 4.68	14/6	9/11	BT + QYYYG	Basic treatment (CCB, ACEI/ARB)	12w	③
Ning (2017)	40/40	45.41 ± 7.59	45.17 ± 6.53	22/18	19/21	BT + QYYYG	Basic treatment (nifedipine)	8w	③
Ding et al. (2015)	80/80	NA	NA	28/32	31/29	BT + QYYYG	Basic treatment (nifedipine, enalapril)	8w	③
Qing (2020)	28/25	60.00 ± 9.59	59.44 ± 10.55	12/16	10/15	BT + QYYYG	Basic Treatment (CCB, ACEI/ARB)	8w	③

^a^ represents the estimation of the sample mean and standard deviation (Luo et al., 2017); T is the experimental group, C is the control group; BT, is the treatment measure for the control group; Outcome indicators: ①Indicators related to vascular elasticity; ②Indicators related to inflammatory factors; ③Blood pressure levels; ④Safety indicators.

### 3.3 Quality evaluation of the included studies

**FIGURE 3:**
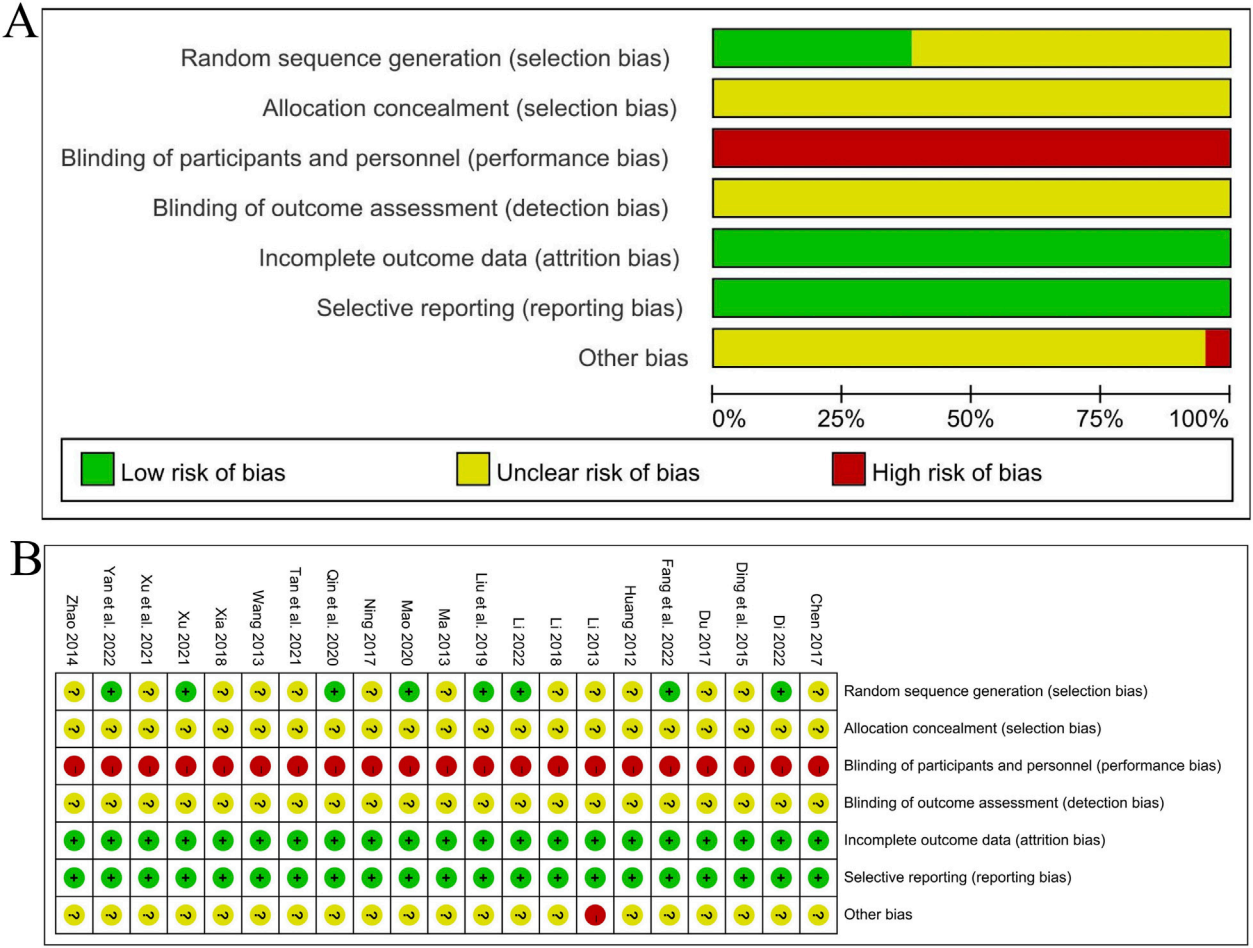
**(A)** Summary of bias risk assessment; **(B)** Detail diagram of bias risk assessment.

The Cochrane Risk of Bias Assessment Tool was used to evaluate from the following seven aspects: 1) Random sequence generation: Five studies (Xu, 2021; Mao, 2020; Yan et al., 2022; Liu et al., 2019; Fang et al., 2022a) indicated that the random number table method was used, and a “low risk of bias” evaluation was made. Three studies (Li, 2022; Dinala Jialiken, 2022; Qin, 2020) indicated that random grouping was carried out using the R statistical software, and a “low risk of bias” evaluation was made. The remaining 13 studies only mentioned the word “random”, and the specific randomization method was not described, so an “unclear risk of bias” evaluation was made; 2) Allocation concealment: None of the 21 studies mentioned allocation concealment, so an “unclear risk of bias” evaluation was made; 3) Blinding of participants and personnel: Although no studies have mentioned it, due to the lack of development of high fidelity traditional Chinese medicine placebos, they have been evaluated as having a “high risk of bias”; 4) Blinding of outcome assessment: None of the studies mentioned it, so all studies were evaluated for “unclear risk of bias”; 5) Incomplete outcome data, 6) Selective reporting: Eight studies (Li, 2022; Dinala Jialiken, 2022; Li, 2018; Xu, 2021; Mao, 2020; Chen, 2017; Fang et al., 2022a; Qin, 2020) had cases of loss to follow-up, but the balance between groups was maintained and the reasons were similar. The outcome data of other studies were complete, and there was no selective reporting of results in all studies, so a “low risk of bias” evaluation was made for all; 7) Other bias: One study (Li, 2013) had a small sample size and an uneven proportion of male and female samples, so a “high risk of bias” evaluation was made. For the other studies, other biases were unknown, and an “unclear risk of bias” evaluation was made. The bias proportion of the included studies is shown in Figure 3A, and the quality evaluation of the included studies is shown in Figure 3B.

### 3.4 Results of meta-analysis

#### 3.4.1 Indicators related to vascular elasticity function

##### 3.4.1.1 PWV-BS

A total of 6 RCT studies (Li, 2022; Dinala Jialiken, 2022; Xu et al., 2021; Tan et al., 2021; Li, 2018; Xia, 2018) reported the situation of PWV-BS of the left and right common carotid arteries after treatment, involving a total of 502 patients, with 249 in the experimental group and 253 in the control group. The heterogeneity of the left common carotid artery (*P* = 0.03, I^2^ = 60%) and the right common carotid artery (*P* = 0.06, I^2^ = 52%) was relatively large. Considering that age has a great influence on the vascular elasticity function of hypertension patients, stratification was carried out according to the average age of each included study (<60 years old, ≥60 years old). After subgroup analysis, the heterogeneity significantly decreased.

In the 3 studies with an average age of ≥60 years old (Tan et al., 2021; Li, 2018; Xia, 2018), the heterogeneity of PWV-BS of the left common carotid artery was small (*P* = 0.90, I^2^ = 0%), and there was a statistically significant difference between groups [MD = −0.58, 95%CI (−0.88, −0.29), *P* < 0.0001]; The heterogeneity of PWV-BS of the right common carotid artery was small (*P* = 0.88, I^2^ = 0%), and there was a statistically significant difference between groups [MD = −0.78, 95%CI (−1.11, −0.45), *P* < 0.00001]. After subgroup analysis, the heterogeneity of different subgroups was significantly reduced, so a fixed-effects model was used for analysis. These two results indicate that in the elderly population, the experimental group with the addition of QYYYG has a better effect in reducing PWV-BS of the left and right common carotid arteries than the control group.

**FIGURE 4:**
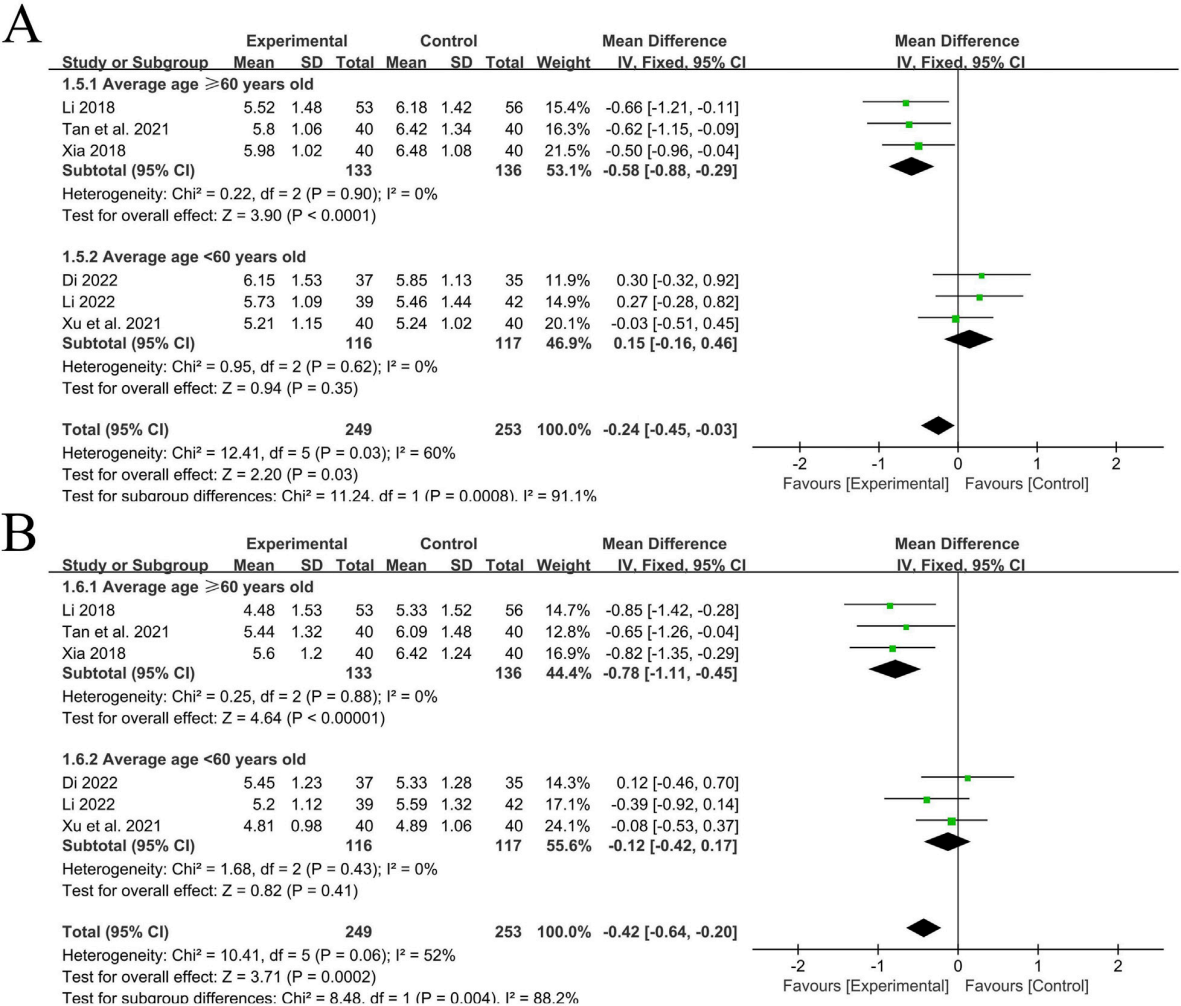
**(A)** PWV-BS of the left common carotid artery; **(B)** PWV‐BS of the right common carotid artery.

In the other 3 studies with an average age of <60 years old (Li, 2022; Dinala Jialiken, 2022; Xu et al., 2021), the heterogeneity of PWV-BS of the left common carotid artery was small (*P* = 0.62, I^2^ = 0%), and there was no statistically significant difference between groups [MD = 0.15, 95%CI (−0.16, 0.46), *P* = 0.35]; The heterogeneity of PWV-BS of the right common carotid artery was small (*P* = 0.43, I^2^ = 0%), and there was no statistically significant difference between groups [MD = −0.12, 95%CI (−0.42, 0.17), *P* = 0.41]. Given the low level of heterogeneity between the two, a fixed-effects model is similarly employed for the analysis. These two results indicate that in the non-elderly population, the effect of the experimental group in reducing PWV-BS of the left and right common carotid arteries is not obvious for the time being, as shown in Figures 4A,B.

##### 3.4.1.2 PWV-ES

A total of 6 RCT studies (Li, 2022; Dinala Jialiken, 2022; Xu et al., 2021; Tan et al., 2021; Li, 2018; Xia, 2018) reported the situation of PWV-ES of the left and right common carotid arteries after treatment, involving a total of 502 patients, with 249 in the experimental group and 253 in the control group. The heterogeneity of the left common carotid artery (*P* = 0.87, I^2^ = 0%) was small, while the heterogeneity of the right common carotid artery (*P* = 0.02, I^2^ = 62%) was relatively large. Subgroup analysis was still carried out according to the average age of each included study (<60 years old, ≥60 years old). After subgroup analysis, the heterogeneity significantly decreased.

In the 3 studies with an average age of ≥60 years old (Tan et al., 2021; Li, 2018; Xia, 2018), the heterogeneity of PWV-ES of the left common carotid artery was small (*P* = 0.99, I^2^ = 0%), and there was a statistically significant difference between groups [MD = −0.79, 95%CI (−1.22, −0.36), *P* = 0.0003]; The heterogeneity of PWV-ES of the right common carotid artery was small (*P* = 0.70, I^2^ = 0%), and there was a statistically significant difference between groups [MD = −1.10, 95%CI (−1.53, −0.66), *P* < 0.00001]. Using a fixed-effects model for analysis, these two results indicate that in the elderly population, the experimental group with the addition of QYYYG has a better effect in reducing PWV-ES of the left and right common carotid arteries than the control group.

**FIGURE 5:**
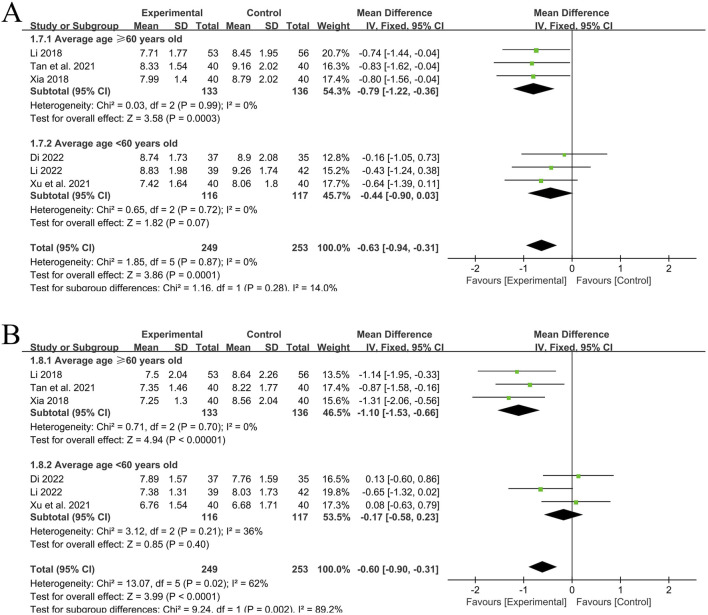
**(A)** PWV-ES of the left common carotid artery; **(B)** PWV‐ES of the right common carotid artery.

In the other 3 studies with an average age of <60 years old (Li, 2022; Dinala Jialiken, 2022; Xu et al., 2021), the heterogeneity of PWV-ES of the left common carotid artery was small (*P* = 0.72, I^2^ = 0%), and there was no statistically significant difference between groups [MD = −0.44, 95%CI (−0.90, 0.03), *P* = 0.07]; the heterogeneity of PWV-ES of the right common carotid artery was small (*P* = 0.21, I^2^ = 36%), and there was no statistically significant difference between groups [MD = −0.17, 95%CI (−0.58, 0.23), *P* = 0.40]. Using a fixed-effects model, these two results indicate that in the non-elderly population, the effect of the experimental group in reducing PWV-ES of the left and right common carotid arteries is not obvious for the time being, as shown in Figures 5A,B.

In conclusion, QYYYG combined with basic treatment can effectively reduce PWV-BS and PWV-ES of the left and right carotid arteries in the elderly population aged ≥60 years old, and has an obvious effect on improving vascular elasticity function. However, there is a significant age distinction. For non-elderly patients aged <60 years old, the effect is not significant for the time being.

#### 3.4.2 Indicators related to inflammatory factors

##### 3.4.2.1 CRP

**FIGURE 6:**
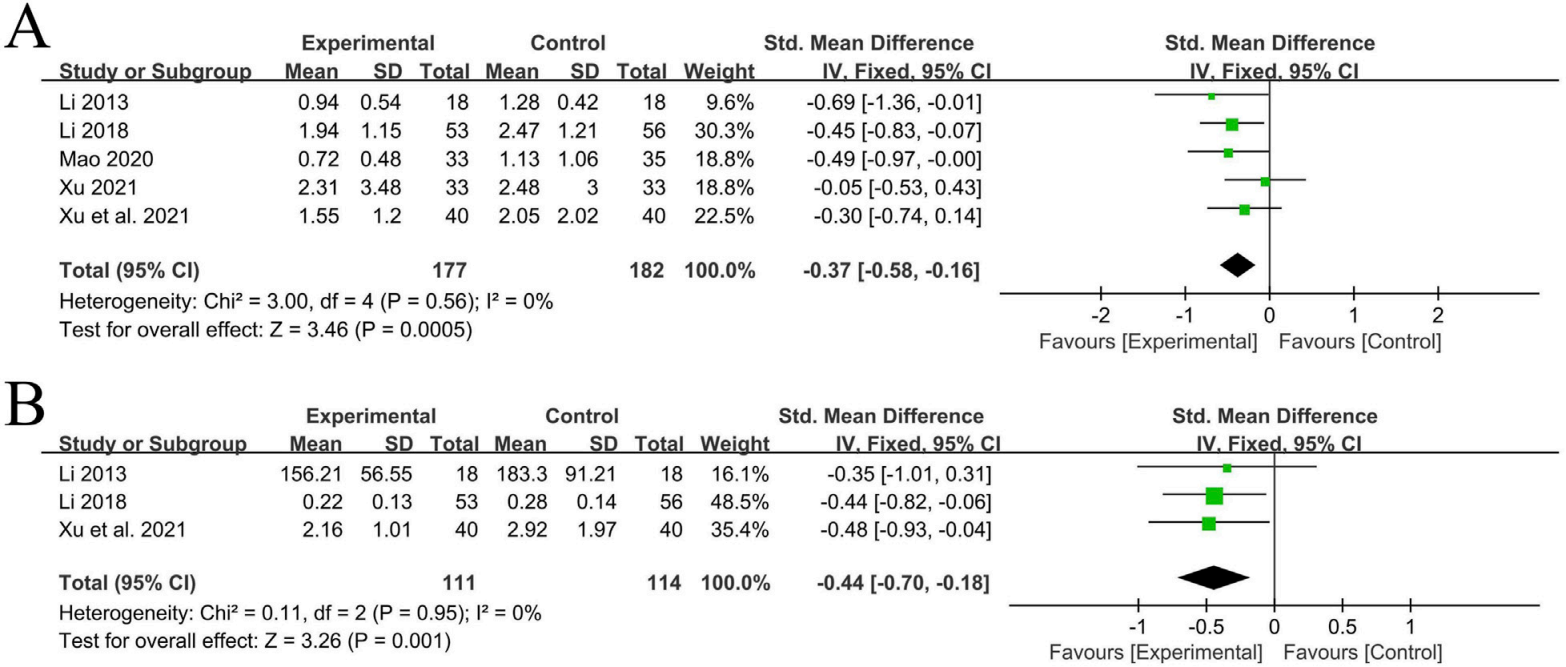
**(A)** Analysis results of CRP; **(B)** Analysis results of TNF‐α.

A total of 5 RCT studies (Xu et al., 2021; Li, 2018; Li, 2013; Xu, 2021; Mao, 2020) reported the situation of CRP after treatment, involving a total of 359 patients, with 177 in the experimental group and 182 in the control group. Due to the different detection methods in the studies, the standardized mean difference (SMD) was used. The heterogeneity among the studies was small (*P* = 0.56, I^2^ = 0%). Analysis using the fixed-effects model showed that the experimental group with the addition of QYYYG had a better effect in reducing CRP than the control group, and there was a statistically significant difference between groups [SMD = −0.37, 95%CI (−0.58, −0.16), *P* = 0.0005], as shown in Figure 6A.

##### 3.4.2.2 TNF-α

A total of 3 RCT studies (Xu et al., 2021; Li, 2018; Li, 2013) reported the situation of TNF-α before and after treatment, involving a total of 225 patients, with 111 in the experimental group and 114 in the control group. Due to the different detection methods and units in the studies, the standardized mean difference was used. The heterogeneity among the studies was small (*P* = 0.95, I^2^ = 0%). Analysis using the fixed-effects model showed that the experimental group with the addition of QYYYG had a better effect in reducing TNF-α than the control group, and there was a statistically significant difference between groups [SMD = −0.44, 95%CI (−0.70, −0.18), *P* = 0.001], as shown in Figure 6B.

#### 3.4.3 Blood pressure levels

##### 3.4.3.1 Systolic blood pressure

**FIGURE 7:**
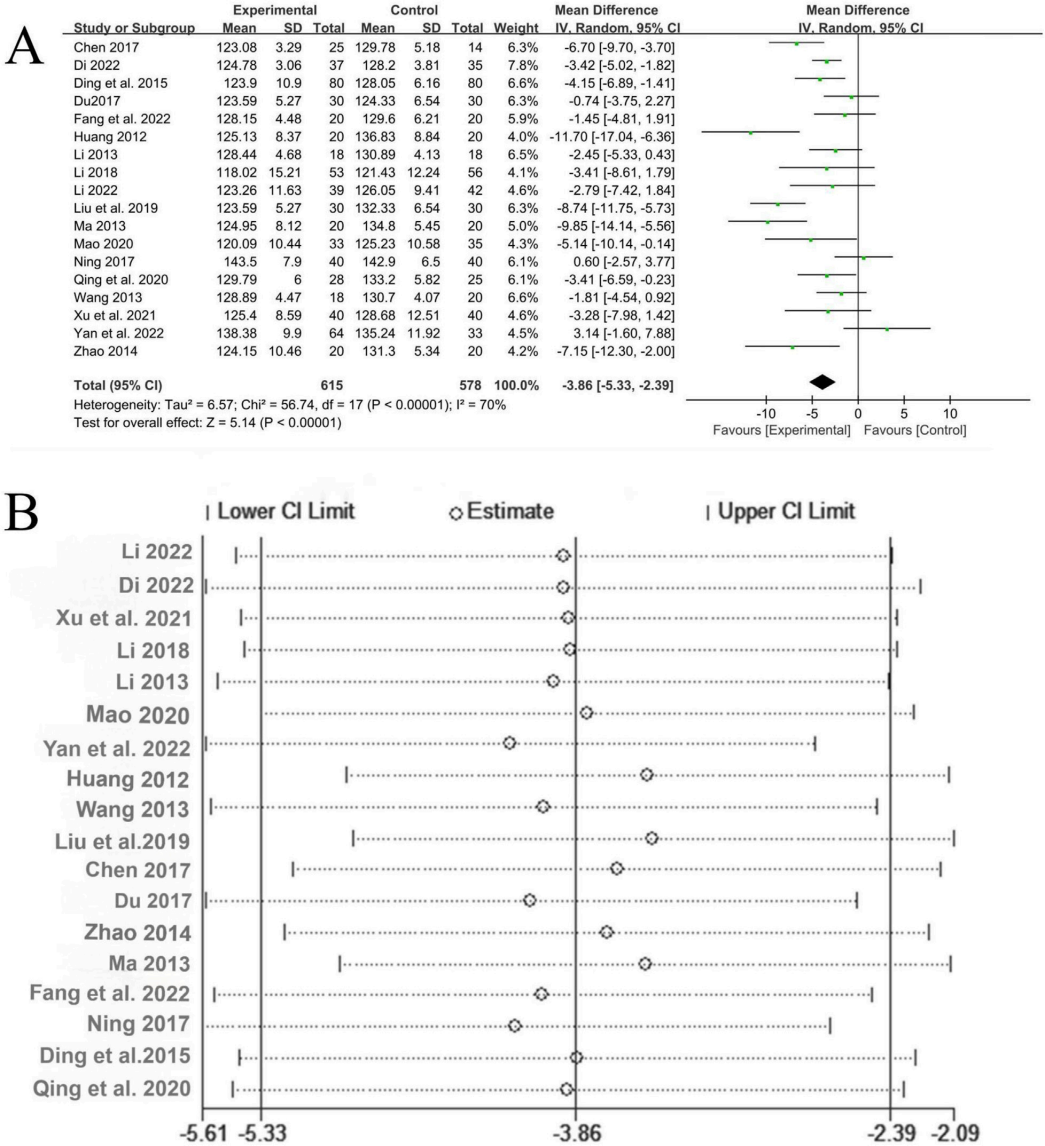
**(A)** Analysis results of systolic blood pressure; **(B)** The sensitivity analysis of systolic blood pressure.

A total of 18 studies (Li, 2022; Dinala Jialiken, 2022; Xu et al., 2021; Li, 2018; Li, 2013; Mao, 2020; Yan et al., 2022; Huang, 2012; Wang, 2013; Liu et al., 2019; Chen, 2017; Du, 2017; Zhao, 2014; Ma, 2013; Fang et al., 2022a; Ning, 2017; Ding et al., 2015; Qin, 2020) reported the treatment situation of systolic blood pressure, involving a total of 1,193 patients, with 615 in the experimental group and 578 in the control group. The heterogeneity among the studies was relatively large (*P* < 0.00001, I^2^ = 70%), so the random-effects model was used for analysis. The Meta-analysis showed that the experimental group with the addition of QYYYG had a better effect in reducing systolic blood pressure than the control group, and there was a statistically significant difference between groups [MD = −3.86, 95%CI (−5.33, −2.39), *P* < 0.00001], as shown in Figure 7A. The sensitivity analysis of systolic blood pressure indicated that after excluding the studies one by one, all the estimated values of the results were still within the 95%CI of the combined effect size, indicating that the overall results had good stability, as shown in Figure 7B.

##### 3.4.3.2 Diastolic blood pressure

**FIGURE 8:**
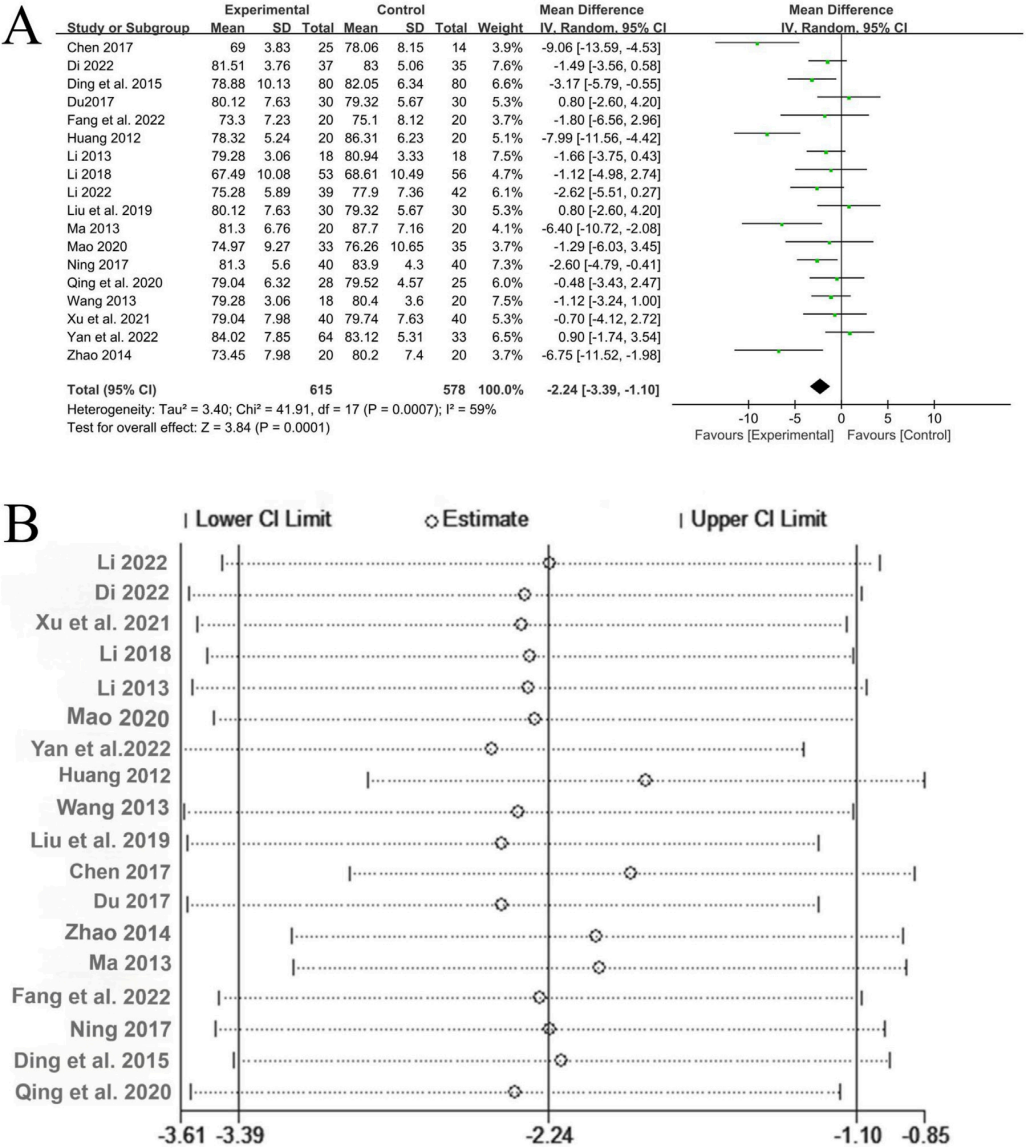
**(A)** Analysis results of diastolic blood pressure; **(B)** The sensitivity analysis of diastolic blood pressure.

A total of 18 studies (Li, 2022; Dinala Jialiken, 2022; Xu et al., 2021; Li, 2018; Li, 2013; Mao, 2020; Yan et al., 2022; Huang, 2012; Wang, 2013; Liu et al., 2019; Chen, 2017; Du, 2017; Zhao, 2014; Ma, 2013; Fang et al., 2022a; Ning, 2017; Ding et al., 2015; Qin, 2020) reported the treatment of diastolic blood pressure, involving a total of 1,193 patients, with 615 in the experimental group and 578 in the control group. There was significant heterogeneity among the studies (*P* = 0.0007, I^2^ = 59%), and the random-effects model was used for analysis. The Meta-analysis showed that the experimental group with the addition of QYYYG had a better effect in reducing diastolic blood pressure than the control group, and there was a statistically significant difference between the groups [MD = −2.24, 95%CI (−3.39, −1.10), *P* = 0.0001], as shown in Figure 8A. The sensitivity analysis of diastolic blood pressure also indicated that the overall results were stable, as shown in Figure 8B.

#### 3.4.4 Safety indicators

**FIGURE 9:**
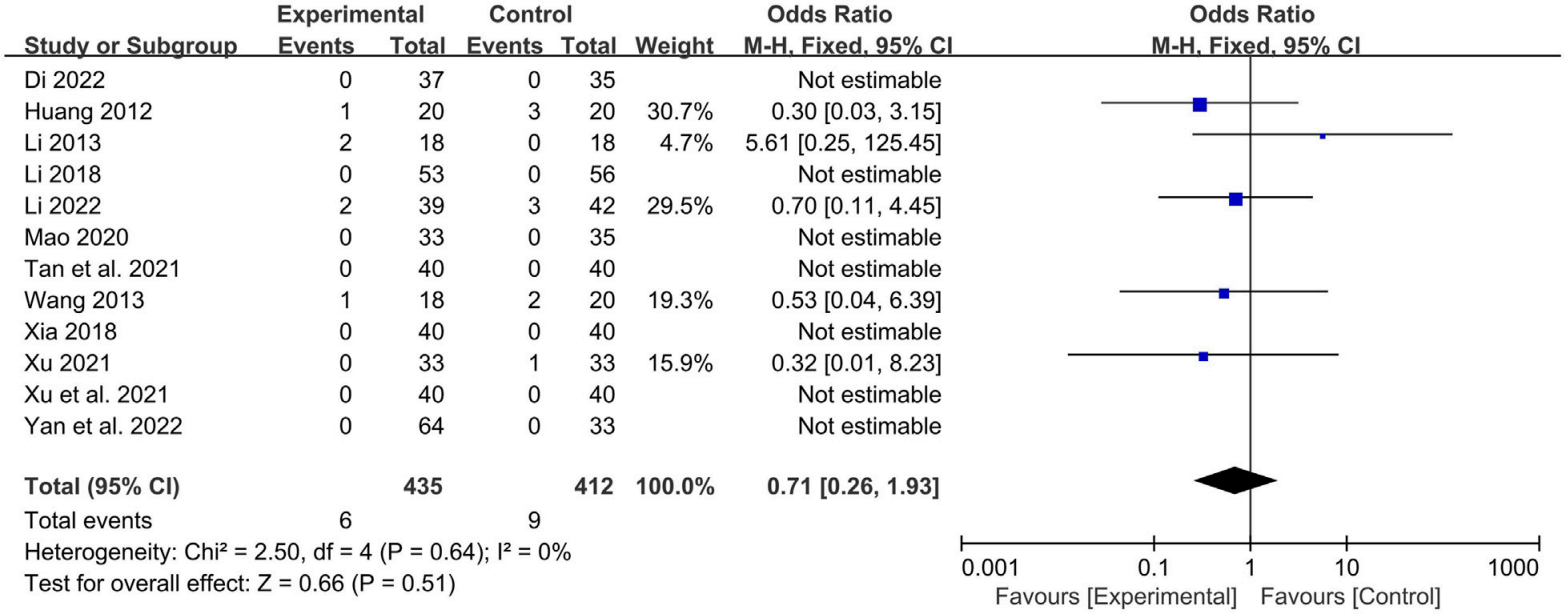
Forest plot of safety indicators.

A total of 12 studies (Li, 2022; Dinala Jialiken, 2022; Xu et al., 2021; Tan et al., 2021; Li, 2018; Xia, 2018; Li, 2013; Xu, 2021; Mao, 2020; Yan et al., 2022; Huang, 2012; Wang, 2013) reported safety analyses, involving a total of 847 patients, with 435 in the experimental group and 412 in the control group. Among them, 7 studies (Dinala Jialiken, 2022; Xu et al., 2021; Tan et al., 2021; Li, 2018; Xia, 2018; Mao, 2020; Yan et al., 2022) clearly reported that no adverse reactions were observed in either the experimental group or the control group. In 5 studies (Li, 2022; Li, 2013; Xu, 2021; Huang, 2012; Wang, 2013), adverse reactions occurred. There were 6 cases in the experimental group, including 3 cases of loose stools, 1 case of mild increase in urine red blood cell count (20 U/L), 1 case of positive urine protein, and 1 case of fever. There were 9 cases in the control group, including 1 case of nausea after taking the medicine, 1 case of positive urine protein, 1 case of elevated alanine aminotransferase (99 U/L), 1 case of elevated uric acid (614 μmol/L), 3 cases of mild headache, and 2 cases of muscle soreness. The heterogeneity among the studies was small (*P* = 0.64, I^2^ = 0%), so a fixed-effects model was used for analysis. Meta-analysis suggested that there was no statistically significant difference in the incidence of adverse reactions between the experimental group with the addition of QYYYG and the control group [OR = 0.71, 95%CI (0.26, 1.93), *P* = 0.51]. This indicates that the addition of QYYYG on the basis of conventional treatment does not increase the adverse reactions of related drugs and has good clinical safety, as shown in Figure 9.

### 3.5 Publication bias

**FIGURE 10:**
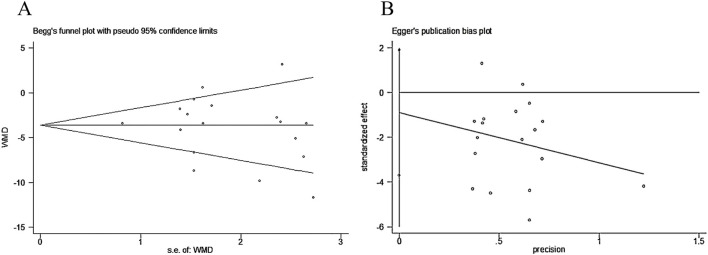
**(A)** Begg’s test; **(B)** Egger’s test.

Among all the outcome indicators, 18 studies reported the systolic blood pressure. Begg’s test (P = 0.306) and Egger’s test (P = 0.505) were performed to evaluate publication bias. Both returned P-values exceeding 0.05, indicating no publication bias was present and confirming the robustness of the results. This is illustrated in Figure 10.

### 3.6 GRADE rating

GRADEpro is used to assess the quality of evidence for different indicators. Among them, the indicators with moderate-quality evidence include: PWV (subgroup), CRP, TNF-α, and safety indicators. The indicators with low-quality evidence are: PWV, systolic blood pressure, and diastolic blood pressure. The detailed GRADE assessment results are presented in Table 2.

**TABLE 2:** GRADE rating.

Quality assessment							No of patients		Effect		Quality	Importance
No of studies	Design	Risk of bias	Inconsistency	Indirectness	Imprecision	Other considerations	T	C	Relative	Absolute		
									(95% CI)			
LCCA: PWV-BS (Better indicated by lower values)												
6	Randomised trials	Serious^a^	Serious^b^	No serious indirectness	No serious imprecision	None	249	253	-	MD 0.24 lower (0.45–0.03 lower)	⊕⊕ΟΟ	CRITICAL
											LOW	
LCCA: PWV-BS(≥60 years old) (Better indicated by lower values)												
3	Randomised trials	Serious^a^	No serious inconsistency	No serious indirectness	No serious imprecision	None	133	136	-	MD 0.58 lower (0.88–0.29 lower)	⊕⊕⊕Ο	CRITICAL
											MODERATE	
LCCA: PWV-BS(<60 years old) (Better indicated by lower values)												
3	Randomised trials	Serious^a^	No serious inconsistency	No serious indirectness	No serious imprecision	None	116	117	-	MD 0.15 higher (0.16 lower to 0.46 higher)	⊕⊕⊕Ο	CRITICAL
											MODERATE	
CRP (Better indicated by lower values)												
5	Randomised trials	Serious^a^	No serious inconsistency	No serious indirectness	No serious imprecision	None	177	182	-	SMD 0.37 lower (0.58–0.16 lower)	⊕⊕⊕Ο	CRITICAL
											MODERATE	
TNF-α (Better indicated by lower values)												
3	Randomised trials	Serious^a^	No serious inconsistency	No serious indirectness	No serious imprecision	None	111	114	-	SMD 0.44 lower (0.7–0.18 lower)	⊕⊕⊕Ο	CRITICAL
											MODERATE	
Systolic blood pressure (Better indicated by lower values)												
18	Randomised trials	Serious^a^	Serious^b^	No serious indirectness	No serious imprecision	None	615	578	-	MD 3.86 lower (5.33–2.39 lower)	⊕⊕ΟΟ	CRITICAL
											LOW	
Diastolic blood pressure (Better indicated by lower values)												
18	Randomised trials	Serious^a^	Serious^b^	No serious indirectness	No serious imprecision	None	615	578	-	MD 2.24 lower (3.39–1.1 lower)	⊕⊕ΟΟ	CRITICAL
											LOW	
Safety indicators												
12	Randomised trials	Serious^a^	No serious inconsistency	No serious indirectness	No serious imprecision	None	6/435	9/412	OR 0.71 (0.26–1.93)	6 fewer per 1,000 (from 16 fewer to 19 more)	⊕⊕⊕Ο	CRITICAL
							(1.4%)	(2.2%)			MODERATE	

^a^ Lack of blinding for participants and personnel.

^b^ High statistical heterogeneity.

### 3.7 Trial sequential analysis

The Trial Sequential Analysis (TSA) was carried out using the TSA 0.9Beta software. The type I error probability was set as α = 0.05, and the statistical power was set as 80%. Taking the sample size as the required information size (RIS), and the horizontal line of Z = 1.96 as the traditional boundary value curve, the TSA was conducted on the data related to the efficacy of vascular damage.

**FIGURE 11:**
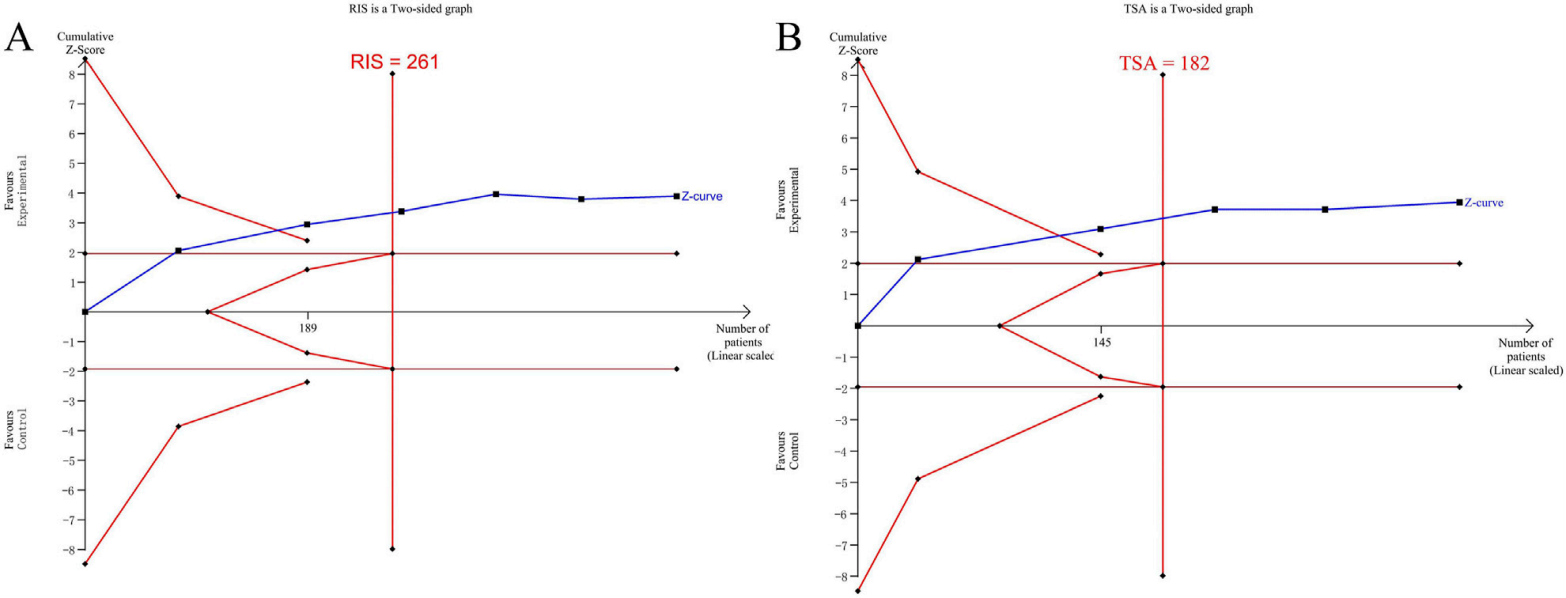
**(A)** TSA analysis of the PWV‐ES value of the left common carotid artery; **(B)** TSA analysis of CRP.

The results of the TSA analysis of the ES value of the left common carotid artery are shown in Figure 11A. The cumulative Z value has successively crossed the traditional boundary value, the TSA boundary value curve, and the RIS line in the first three studies. The results of the TSA analysis of peripheral blood CRP are shown in Figure 11B. It also crossed the traditional boundary value and the TSA boundary value curve, and crossed the RIS line in the third study.

The TSA analysis indicates that the included sample size has reached the sample size required for the Meta-analysis. There is no need for more clinical trials, and it has been definitely concluded that the addition of QYYYG has a better efficacy in reducing the PWV-ES value of the common carotid artery and inflammatory factors.

## 4 Discussion

### 4.1 Hypertension research in TCM

According to the China Hypertension Survey (CHS), it is estimated that the crude prevalence rate of hypertension among adult residents in China from 2012 to 2015 was 27.9% (Wang et al., 2018), which seriously endangers public health. Modern research shows that traditional Chinese medicine has important advantages in preventing and treating hypertension in terms of enhancing efficacy and reducing side effects, multi-target effects, and long-term stability (Liu et al., 2015).

QYYYG are composed of six kinds of medicinal herbs, namely, Herba Bidentis Bipinnatae, Scrophulariae Radix, Alismatis Rhizoma, Polygoni Multiflori Radix, Corni Fructus, Cyathulae Radix (Fan et al., 2025). With three tonifying and three purging herbs, it can achieve the effects of tonifying the healthy qi without leaving the pathogenic factors and purging the pathogenic factors without harming the healthy qi (Zou et al., 2017). The whole formula combines the functions of attacking and tonifying, harmonizes yin and yang, makes the blood vessels unobstructed, dissipates the heat-toxin, and nourishes the burned blood vessels. Current clinical trials have demonstrated that QYYYG exhibits particularly prominent therapeutic effects in elderly populations. Specifically, QYYYG not only reduces the coefficient of blood pressure variability across all time periods in elderly patients (Ni et al., 2016) but also ameliorates early renal damage and renal fibrosis (Fang et al., 2022a). Additionally, it can delay the aging process by enhancing energy metabolism, mitigating DNA oxidative damage, and maintaining the balance of inflammatory factors (Fang et al., 2025). These unique effects in the elderly population are closely associated with age-related physiological changes, including decreased vascular elasticity, increased arterial stiffness, neurohumoral regulatory imbalance, and the accumulation of multiple risk factors.

Clinical pharmacological studies have demonstrated that QYYYG not only effectively controls blood pressure but also ameliorates target organ damage in hypertension. Specifically, QYYYG blocks the activation of the NLRP3 inflammasome by enhancing Nrf2 signaling and inhibiting myocardial inflammation, oxidative stress, and pyroptosis, thereby effectively improving cardiac remodeling in hypertensive patients (Xu et al., 2025). Additionally, QYYYG exerts protective effects against hypertensive renal injury through multiple mechanisms, including regulation of the TRPC6-CaMKKβ-AMPK-mTOR pathway (Ma et al., 2023), modulation of the HIF-1α/PKM2 positive feedback loop (Qian et al., 2021), and epigenetic mechanisms associated with nicotinamide N-methyltransferase (NNMT) expression (Zhang et al., 2020).

High-quality randomized controlled trials (RCTs) are essential for clinical medical research. However, due to the inherent characteristics of traditional Chinese medicine (TCM), insufficient methodological awareness among researchers, considerations of implementation costs, and clinical ethical requirements, current RCTs on TCM for hypertension are deficient in both scale and quality. Notably, deficiencies in blinding are particularly prominent, manifesting in inadequate allocation concealment, incomplete blinding of participants and researchers, and lack of blinding in outcome assessment. To address these limitations, future efforts should focus on: 1) Developing high-fidelity TCM placebos (e.g., bionic technology to simulate appearance and odor); 2) Optimizing outcome measures (increasing objective indicators); 3) Exploring “adaptive designs” (retaining moderate individualization while maintaining standardization). These strategies may progressively improve the methodological rigor of TCM clinical trials.

### 4.2 Summary of results

The Meta-analysis shows that the combination of conventional treatment methods and QYYYG in the treatment of hypertensive vascular damage has obvious value in improving vascular elasticity, reducing inflammatory response, and lowering blood pressure. Moreover, it does not increase the related adverse drug reactions and has good clinical application safety. In this study, the results of the subgroup analysis of PWV in the left and right common carotid arteries indicate that QYYYG have different degrees of influence on the vascular elasticity of hypertensive patients of different age groups. This conclusion suggests that when studying the treatment of hypertensive vascular damage, more attention should be paid to the baseline level of the average age of patients.

The application of trial sequential analysis overcomes the limitations of traditional Meta-analysis, reduces false positive results caused by random errors, and estimates the RIS value to provide a termination criterion for clinical trials (Xia et al., 2013). After analysis and correction, there is conclusive evidence that the addition of QYYYG can reduce the PWV-ES and peripheral blood CRP. There is no need to include more studies to verify this conclusion.

### 4.3 Advantages and limitations

The advantages of this study are as follows. Firstly, by centering on the renowned prescriptions that are extensively applied in clinical practice, a meta-analysis was conducted on controversial issues from the perspective of vascular damage. Secondly, the integrated application of meta-analysis and sequential analysis furnishes the study with the statistical significance threshold and the ineffectiveness threshold of the intervention effect. Thirdly, subgroup analysis revealed the impact of age and other factors on the efficacy of the drug. Fourthly, the heterogeneity of the included indicators is basically low, and the conclusion is credible.

This study still has some limitations. On the one hand, all the retrieved literature are in Chinese, and there are no English articles, which may lead to bias. On the other hand, the included studies carry a risk of bias, which reduces the credibility of the evidence. Notably, due to limitations in the current state of TCM-related research, none of the trials provided information on blinding procedures. Additionally, most existing studies on QYYYG have used endpoint values, while neglecting the change values compared with baseline. Change values can better reflect therapeutic effects and minimize biases caused by baseline heterogeneity. Future researchers should pay attention to these issues.

## 5 Conclusion

Our research findings indicate that the integration of QYYYG with basic treatment demonstrates a superior capacity in ameliorating mild-to-moderate hypertension and vascular damage. However, the outcomes of our study are circumscribed by the sub-optimal quality of the methodology and evidence. As a result, there is a continuing requirement for a greater number of high-quality, multicenter, large-scale sample investigations to validate these results.

## Data Availability

The raw data supporting the conclusions of this article will be made available by the authors, without undue reservation.
