# Supplementary material for: Qianyang Yuyin Granules for vascular damage in mild-to-moderate hypertensive patients: a systematic review with meta-analysis and trial sequential analysis
Source: Front Pharmacol. 2025 Nov 19;16:1612508. doi: 10.3389/fphar.2025.1612508 (PMC12672466; doi:10.3389/fphar.2025.1612508)
Supplement: Supplementary file 1 [file DataSheet1.docx]

**Retrieval Strategy**

**1 Individual retrieval**

Using “Hypertension” “Qianyang Yuyin Granule” as the key words for individual retrieval, we found that the keywords for literature retrieval were as follows: “Qianyang Yuyin Granule*” “Hypertension” “Hypertensive Patient*” “Hypertensive Disease” “Hypertensive Disorder*”.

**2 AI-assisted retrieval**

Through individual retrieval of literature, problems such as incomplete literature and inconsistent themes were found, which are related to the different expressions of "hypertension" and "Qianyang Yuyin granules". Therefore, we combine the large language model to carry out AI-assisted retrieval. Adopt the retrieval keywords obtained by individual retrieval, and ask the DeepSeek-V3 language model according to different databases. It is required to synthesize the recall rate and precision rate, generate the specific logical relationship and retrieval formula, and use the recommended retrieval formula for database retrieval. Finally, the retrieved documents are imported into Zotero document management software for management and analysis. The specific reference process is shown in Figure S1 and the database list is shown in Table S1.


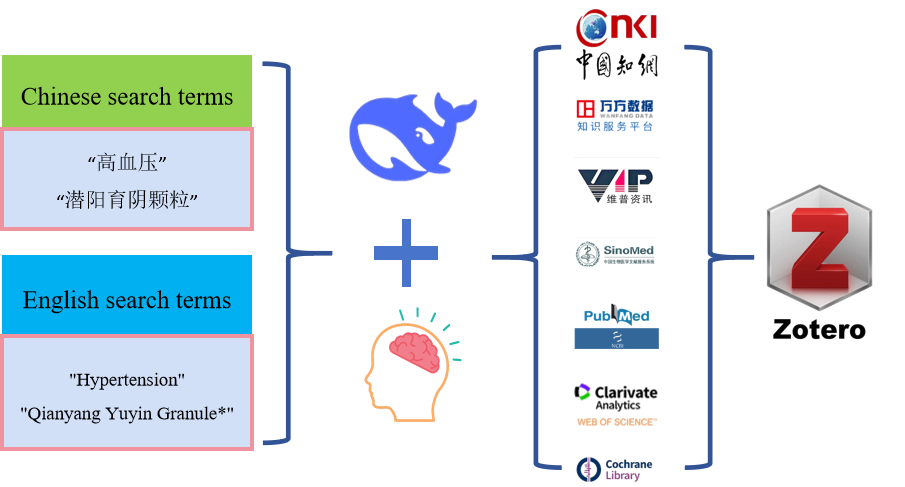


Figure S1 AI-assisted retrieval flow chart

Table S1 Database list

| Database Name | Database Address |
| --- | --- |
| CNKI | <https://www.cnki.net> |
| Wanfang Data | https://www.wanfangdata.com.cn/ |
| VIP | https://qikan.cqvip.com/ |
| CBM | http://www.sinomed.ac.cn/ |
| PubMed | https://pubmed.ncbi.nlm.nih.gov/ |
| Web of Science | http://www.webofscience.com |
| Cochrane Library | www.cochranelibrary.com/ |

**2.1 CNKI**


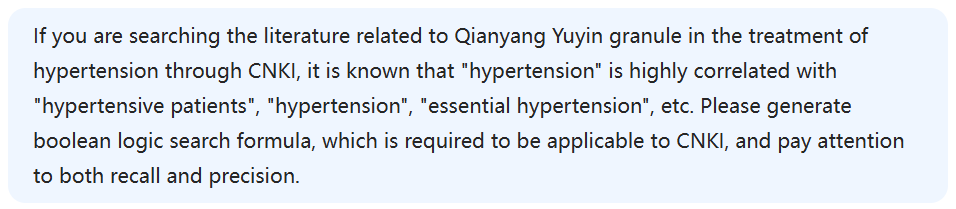


Figure S2 Generating relevant Boolean logic retrieval formula for CNKI based on DeepSeek-V3

Combined with the above results, the retrieval formula is generated through deep thinking of DeepSeek-V3:

(SU=('高血压' + '高血压患者' + '高血压病' + '原发性高血压')) AND SU=('潜阳育阴颗粒')

Through "professional search", check "Chinese and English expansion", and limit it to "academic journals" and "dissertations". A total of 60 search results were obtained.


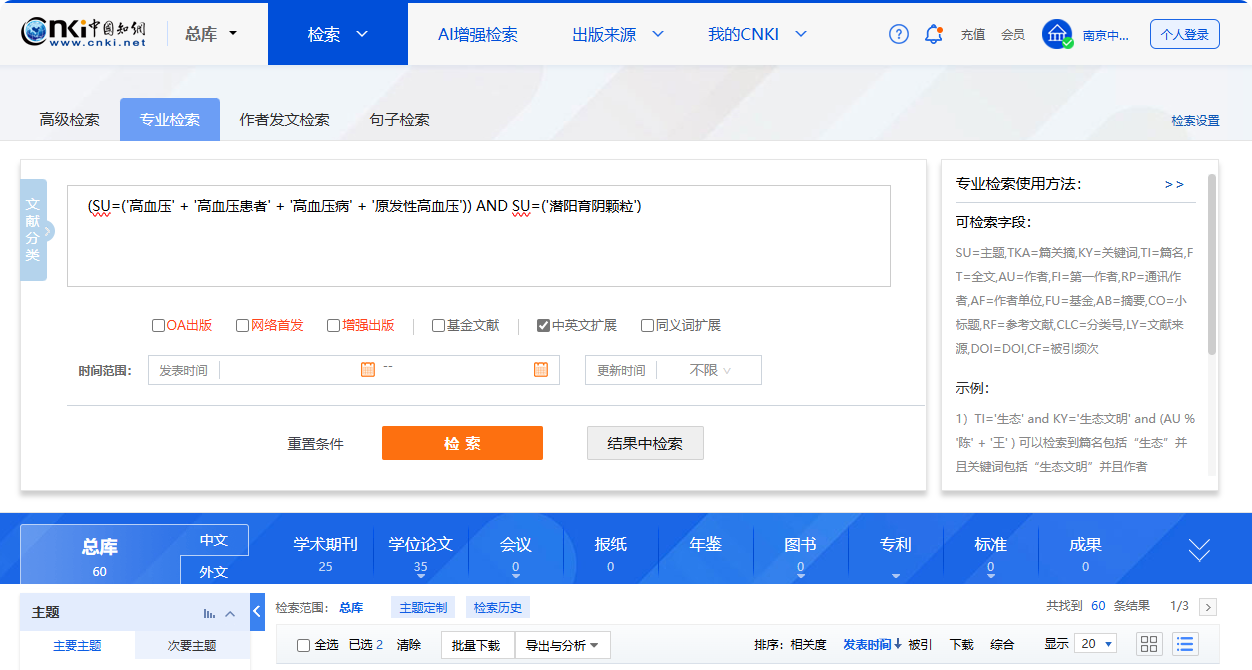


Figure S3 Professional retrieval based on CNKI

**2.2 Wanfang Data**


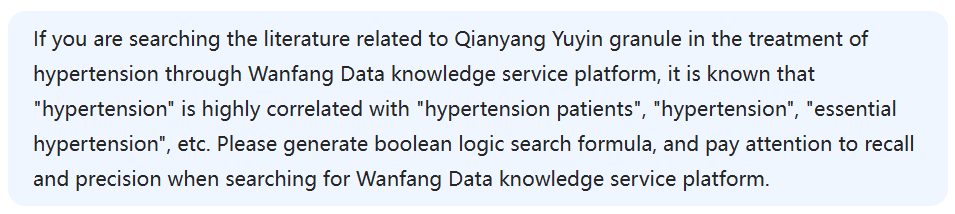


Figure S4 Generating relevant Boolean logic retrieval formula for Wanfang Data based on DeepSeek-V3

Combined with the above results, the retrieval formula is generated through deep thinking of DeepSeek-V3:

("潜阳育阴颗粒" OR "潜阳育阴") AND ("高血压" OR "高血压患者" OR "高血压病" OR "原发性高血压")

Through "professional search", check "Chinese and English expansion" and "subject word expansion", which are limited to "academic journals" and "dissertations", and a total of 39 search results are obtained.


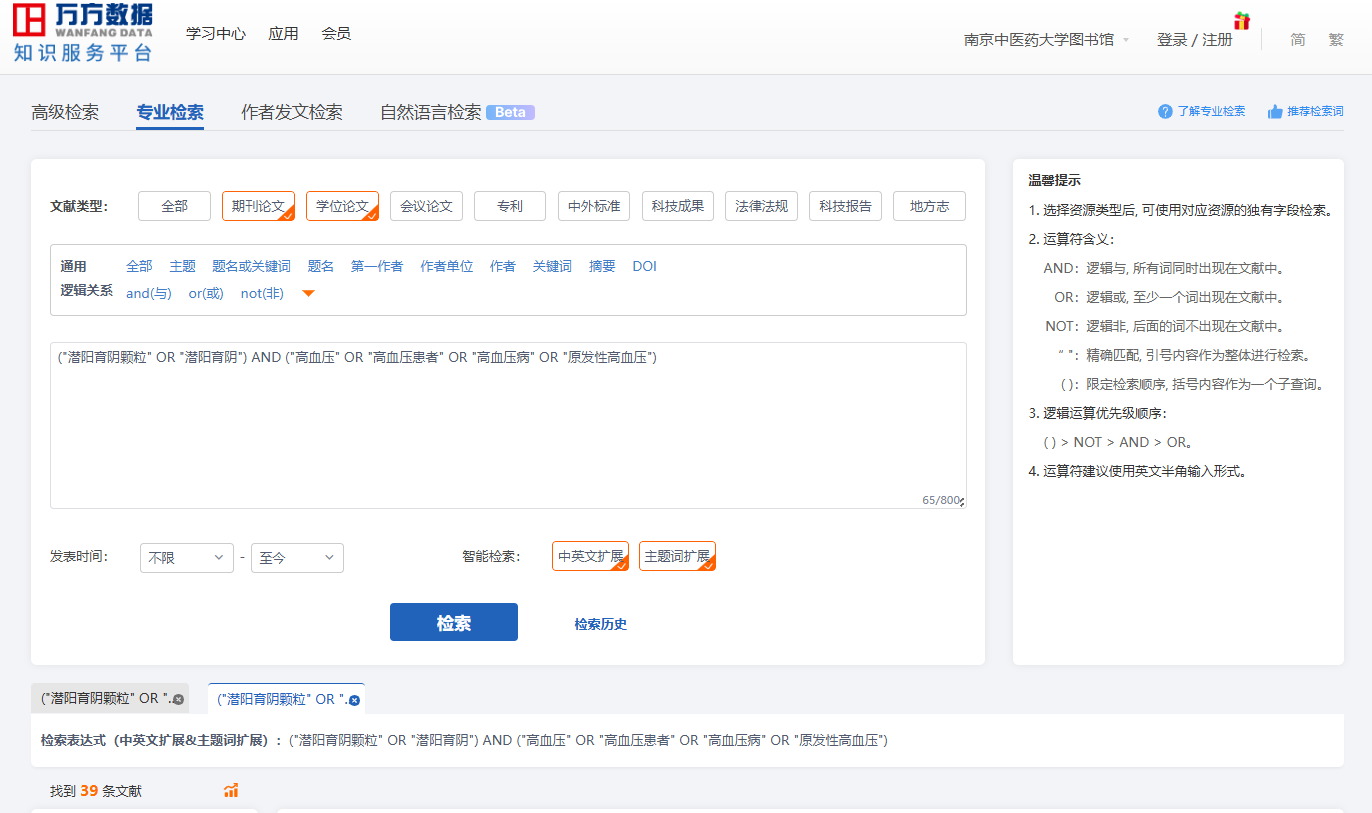


Figure S5 Professional retrieval based on Wanfang Data

**2.3 VIP**


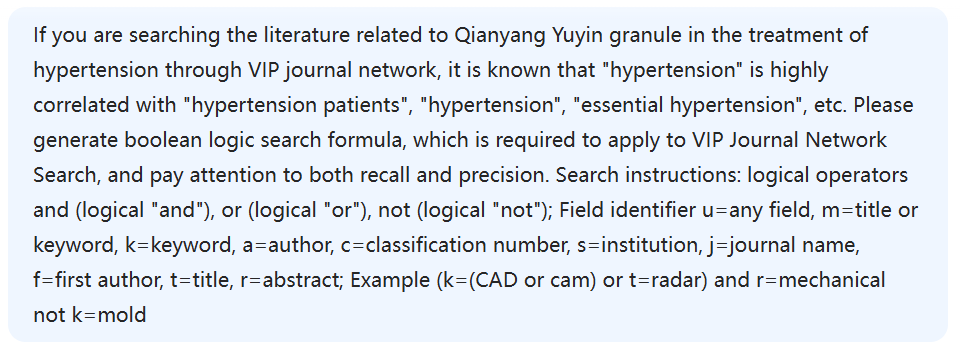


Figure S6 Generating relevant Boolean logic retrieval formula for VIP based on DeepSeek-V3

Combined with the above results, the retrieval formula is generated through deep thinking of DeepSeek-V3:

(M=(高血压 OR 高血压患者 OR 高血压病 OR 原发性高血压)) AND (M=潜阳育阴颗粒 OR R=潜阳育阴颗粒)

A total of 24 search results were obtained through "retrieval search".


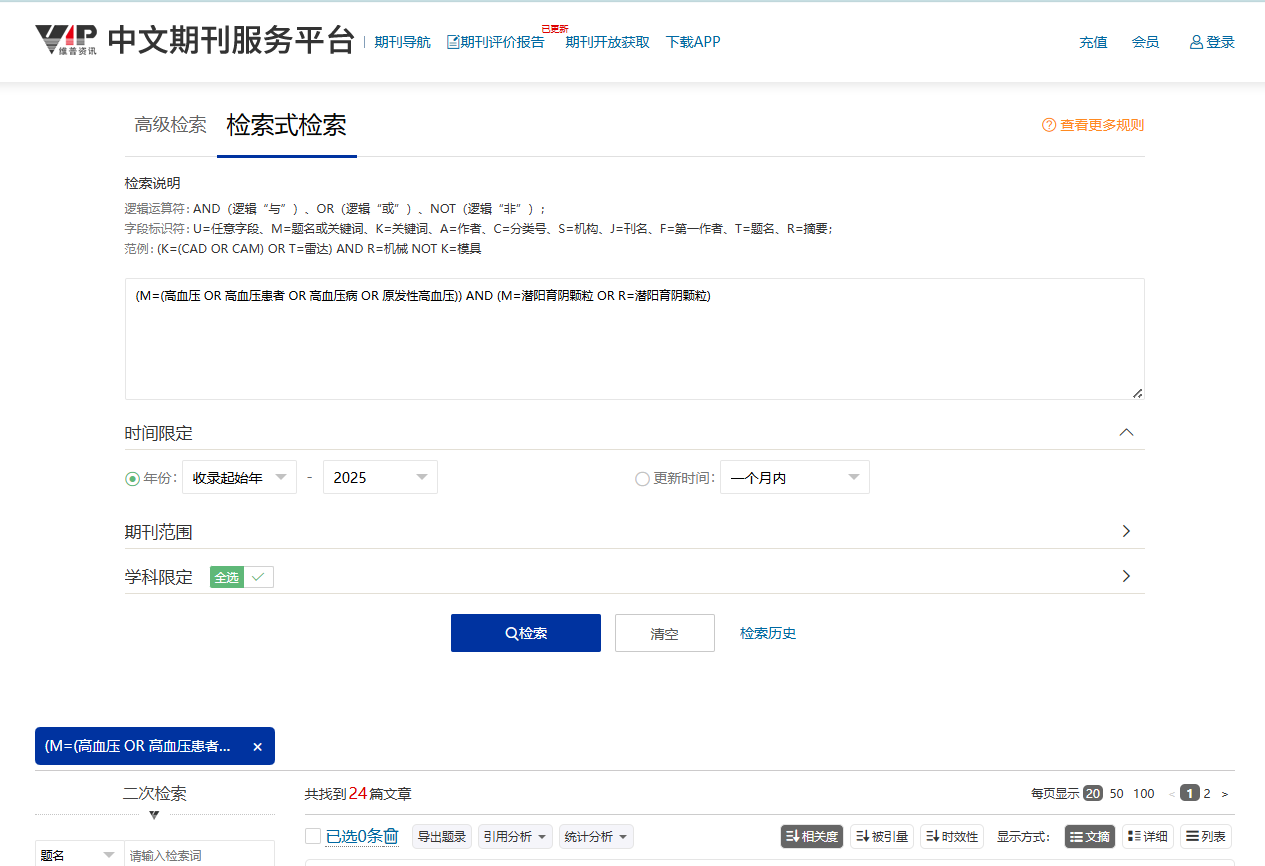


Figure S7 Professional retrieval based on VIP

**2.4 CBM**


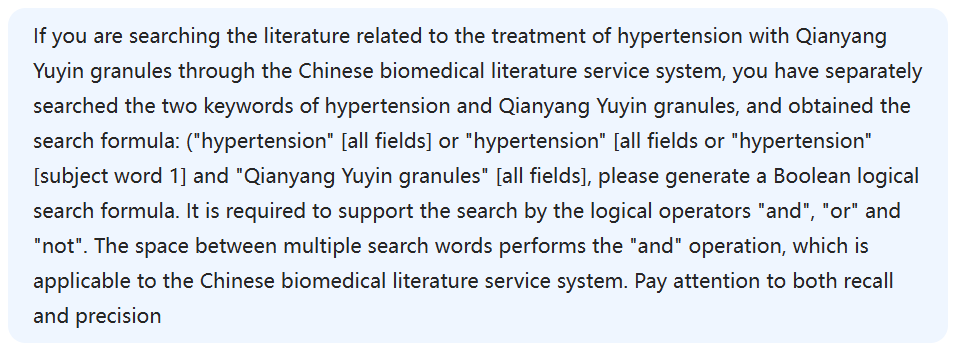


Figure S8 Generating relevant Boolean logic retrieval formula for CBM based on DeepSeek-V3

(1) The key word "hypertension" was retrieved by advanced search, and the detailed search expression was obtained: ("高血压"[全部字段] OR "Hypertension"[全部字段] OR "高血压"[主题词])。

(2) By using advanced search, the keyword "Qianyang Yuyin granules" was searched, and the detailed search expression was obtained: "潜阳育阴颗粒"[全部字段]。

(3) According to the retrieval expression obtained from two separate searches, the key words are adjusted through deep thinking to generate the retrieval formula:

(("高血压"[全部字段] OR "Hypertension"[全部字段] OR "高血压"[主题词])) AND ("潜阳育阴颗粒"[全部字段])

Finally, through the "advanced search", a total of 26 search results were obtained.


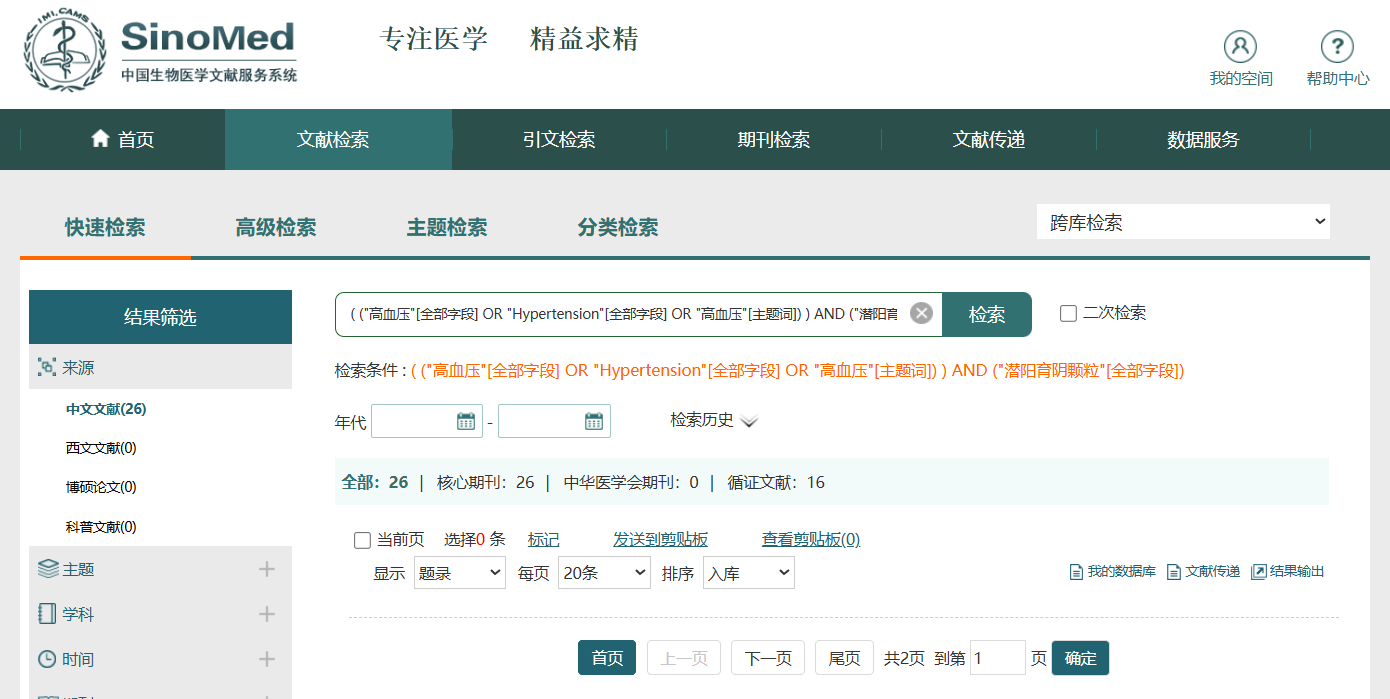


Figure S9 Professional retrieval based on CBM

**2.5 PubMed**

(1) Select the "mesh database" database under the home page of the official website to determine the search subject words. Enter the word "hypertension", find the corresponding subject word "hypertension", include its synonyms in "entry terms", and determine "hypertension" as the subject word, and "blood pressure, high" "high blood pressure" "high blood pressure" as the sub subject word.


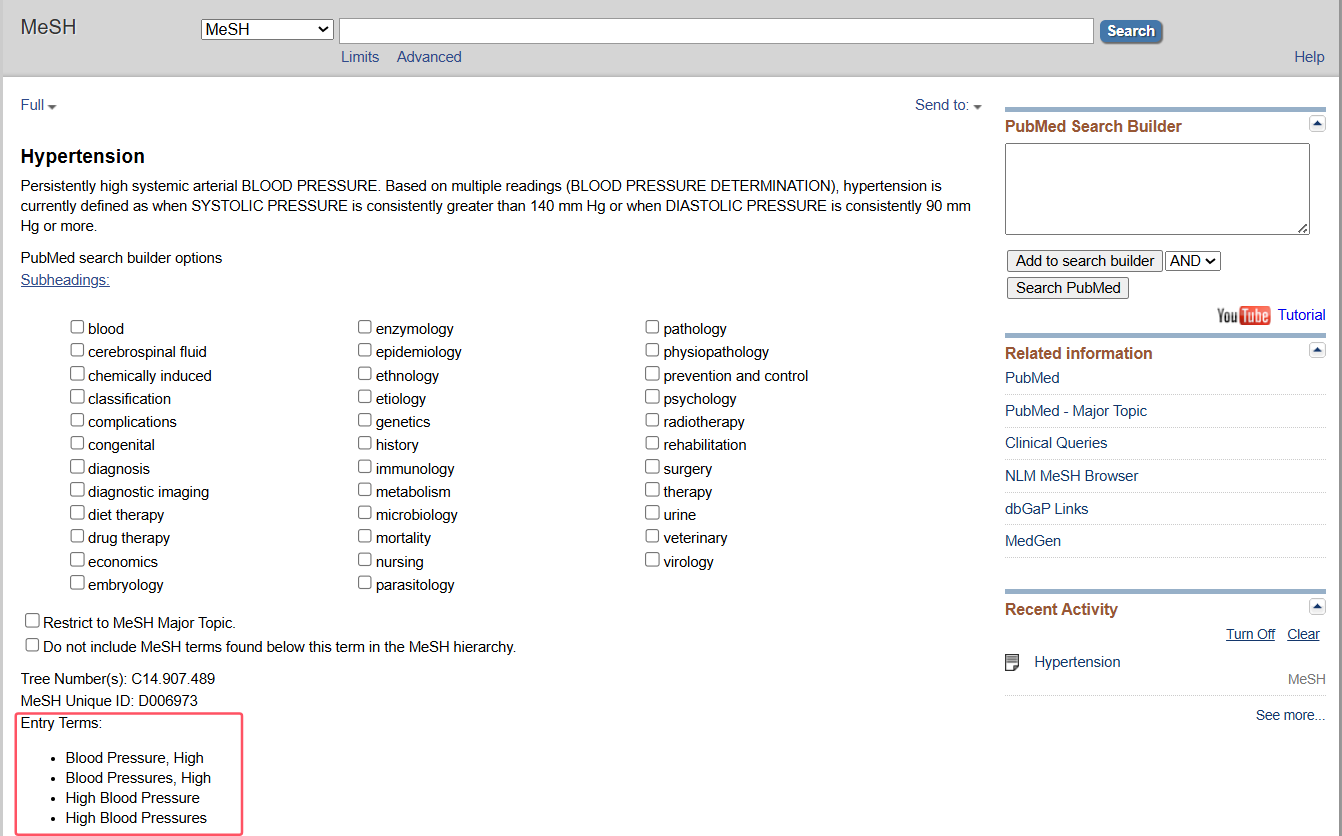


Figure S10 Adjustment of key words in PubMed database

(2) After determining the subject words, think deeply through DeepSeek-V3, and the retrieval expression is as follows:

("Hypertension"[Mesh] OR ( ("blood pressure"[tiab] OR "blood pressures"[tiab]) AND ("high"[tiab] OR "elevated"[tiab] OR "increased"[tiab]) ) OR "High Blood Pressure"[tiab] ) AND ( "Qianyang Yuyin"[tiab] OR "Qian Yang Yu Yin"[tiab] OR "Qian-Yang-Yu-Yin"[tiab] OR ("qyyy"[tiab] AND ("granule*"[tiab] OR "particle*"[tiab]))) NOT (("animal"[tiab] OR "mice"[tiab] OR "rat"[tiab]) NOT "humans"[Mesh])


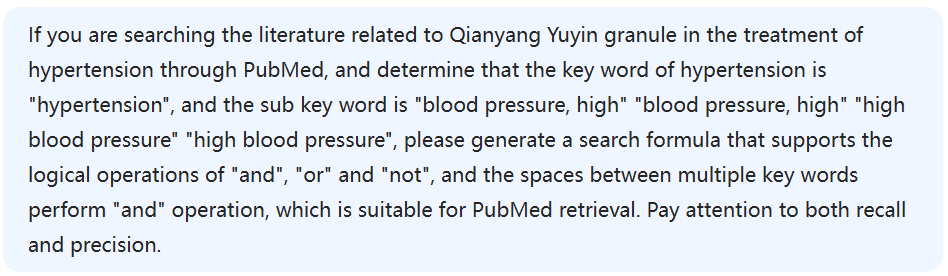


Figure S11 Generating relevant Boolean logic retrieval formula for PubMed based on DeepSeek-V3

Through "advanced search", a total of 5 search results were obtained.


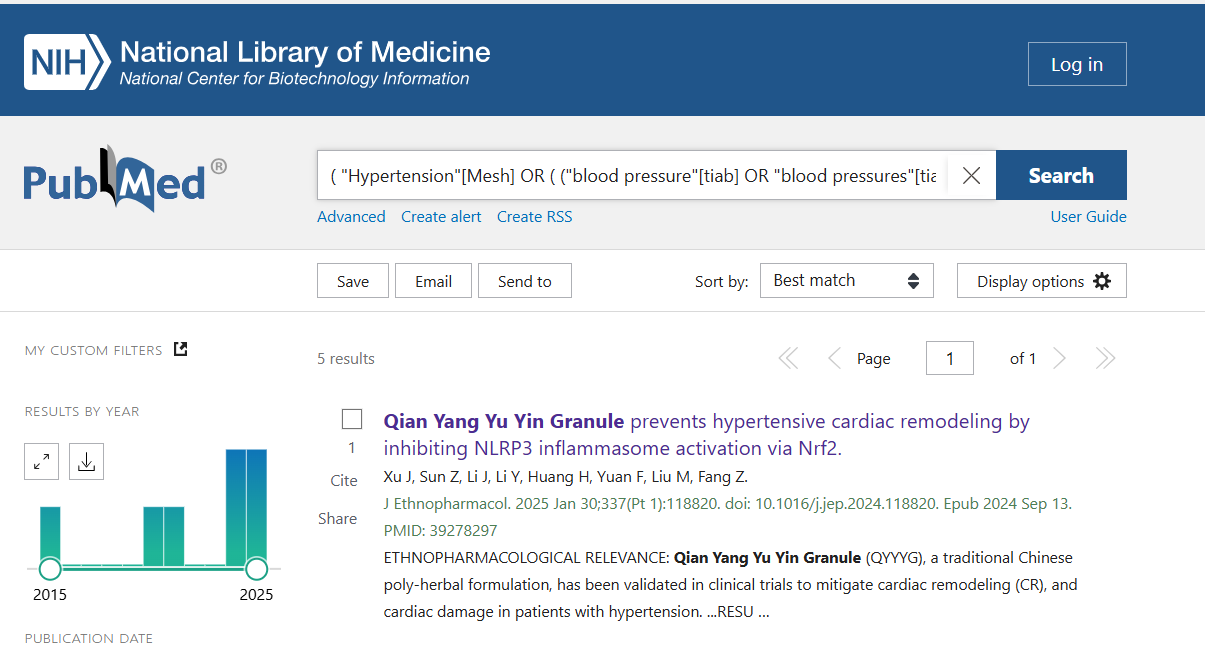


Figure S12 Professional retrieval based on PubMed

**2.6 Web of Science**


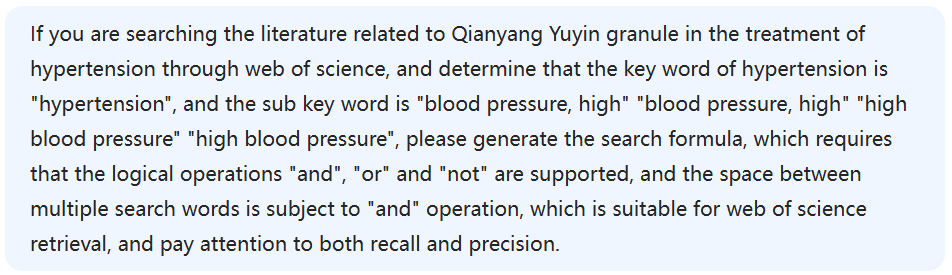


Figure S13 Generating relevant Boolean logic retrieval formula for Web of Science based on DeepSeek-V3

Through deep thinking of DeepSeek-V3, the key words are expanded to form a search type:

TS=((Hypertension OR "Blood Pressure, High" OR "Blood Pressures, High" OR "High Blood Pressure" OR "High Blood Pressures") AND (Qianyang Yuyin* OR "Qianyang Yuyin Granule" OR "Qianyang Yuyin Granules" OR "Qianyang Yuyin Keli")) NOT TS=((diabetes OR "myocardial infarction"))

Through the "retrieval search", a total of 7 search results were obtained.


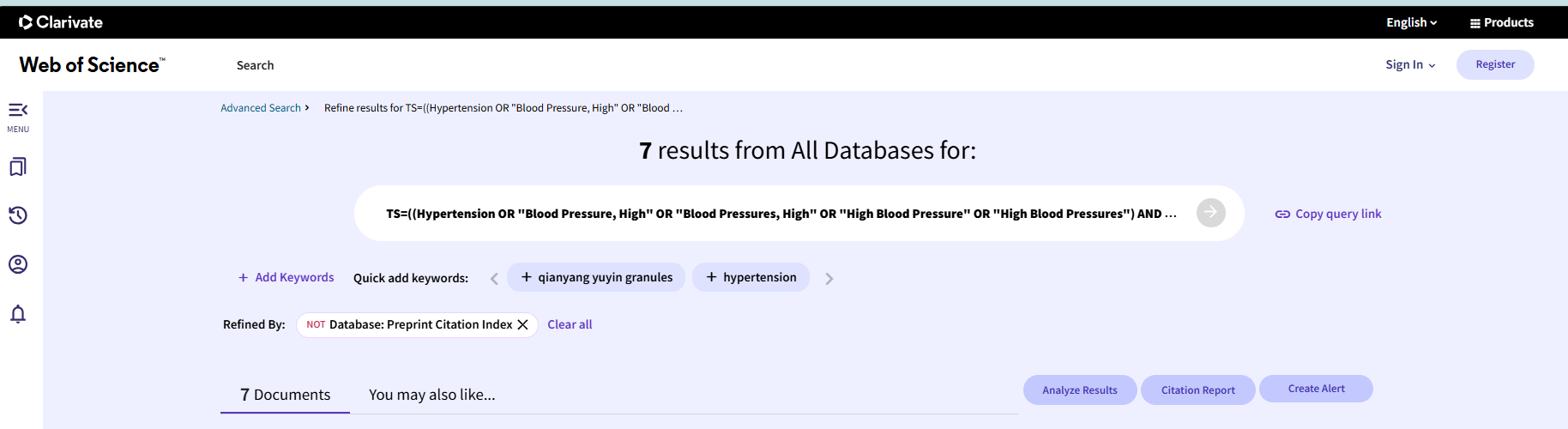


Figure S14 Professional retrieval based on Web of Science

**2.7 Cochrane Library**


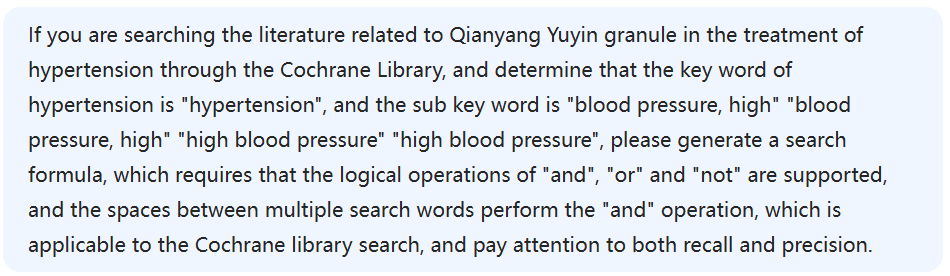


Figure S15 Generating relevant Boolean logic retrieval formula for Cochrane Library based on DeepSeek-V3

Through deep thinking of DeepSeek-V3, the key words are expanded to form a search type:

(Hypertension OR "Blood Pressure, High" OR "Blood Pressures, High" OR "High Blood Pressure" OR "High Blood Pressures") AND ("Qianyang Yuyin Granule" OR "Qianyang Yuyin" OR "Qian Yang Yu Yin" OR "QYYY Granule")

Through the "advanced search", a total of 3 search results were obtained.


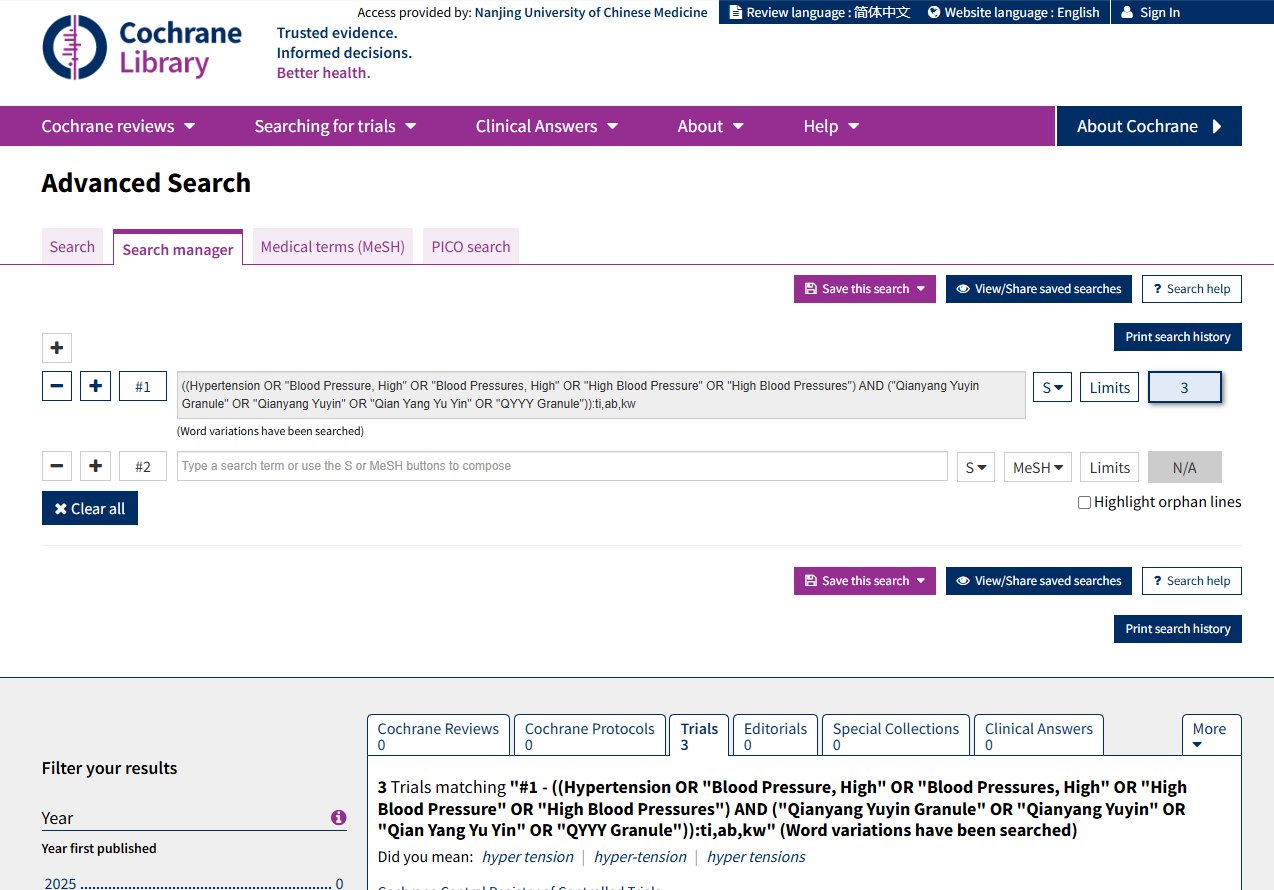


Figure S16 Professional retrieval based on Cochrane Library
